# Intramolecularly Labeled Reference Standards of Sulfamethoxazole for Fragment-Specific Isotope Analysis by Electrospray Ionization Orbitrap Mass Spectrometry

**DOI:** 10.1021/jasms.5c00402

**Published:** 2026-02-06

**Authors:** Aoife Canavan, Christopher Dirr, Martin Elsner

**Affiliations:** TUM School of Natural Sciences, Department of Chemistry, Chair of Analytical Chemistry and Water Chemistry, 9184 Technical University of Munich, Lichtenbergstraße 4, 85748 Garching, Germany

**Keywords:** isotope standards, intramolecular isotope ratios, reference materials, kinetic isotope effect, isotope fractionation, Orbitrap-IRMS, collision-induced fragmentation, antibiotics, sulfonamides

## Abstract

The widespread presence of pharmaceuticals, including antibiotics, in our aquatic environment raises important societal concerns. When studying their environmental fate, stable isotope analysis of nitrogen and carbon at natural abundance offers unique insight into source fingerprinting and degradation-associated kinetic isotope effects. Here, we synthesized compound-specific reference standards to enable electrospray ionization (ESI) Orbitrap mass spectrometry (MS) for fragment-specific carbon and nitrogen isotope analysis (Δδ^13^C and Δδ^15^N) of sulfamethoxazole (SMX), a most frequently detected antibiotic. Fragment-specific isotope analysis relied on fragmentation of SMX ions in the collision cell, resulting in two fragment ions representing the aniline part (*m*/*z* = 92, F92) and the 3-amino-5-methylisoxazole ring (*m*/*z* = 99, F99) of SMX. Reference materials were prepared (i) through total synthesis of SMX from labeled precursors that resulted in specific positions labeled with ^13^C and ^15^N, (ii) followed by the mixing of labeled SMX with SMX at natural abundance. The bulk isotope values of these in-house standards were determined by elemental analysis isotope ratio mass spectrometry and used for calibration of the ESI-Orbitrap-MS method. Injecting standards directly into the ESI-Orbitrap-MS resulted in 95% confidence intervals (CIs) of 0.7‰ and 3.4‰ for Δδ^13^C and Δδ^15^N in F92, respectively, and 1.3‰ and 2.9‰ for Δδ^13^C and Δδ^15^N in F99, for quintuplicate measurements of standards. A proof-of-principle demonstration shows that this approach could indeed successfully quantify changes in fragment-specific isotopic signatures, Δδ^13^C and Δδ^15^N, during degradation of SMX.

## Graphical abstract

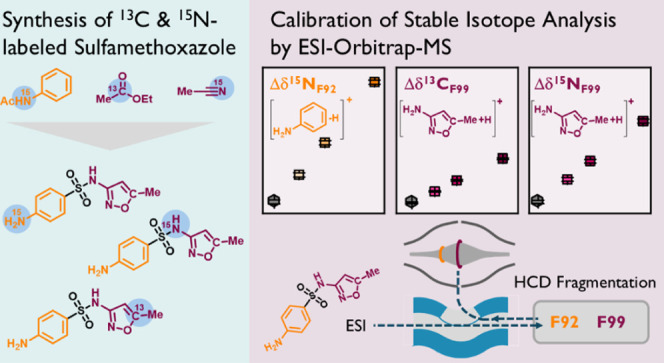

## Introduction

The contamination of aquatic systems with pharmaceuticals has become a growing environmental concern, driven by the increasing detection of these compounds in water bodies worldwide.^1,2^ Among them, sulfamethoxazole (SMX) stands out, a synthetic antibiotic that is abundantly used in both human and veterinary medicine, and which has gained attention due to its persistence and possible ecological risks.^3–6^ Although usually present in low concentrations, antibiotics like SMX can foster the development of antibiotic resistance genes.^7^ Since SMX is not fully metabolized in humans or animals, it enters the environment through livestock farming, aquaculture effluents, and wastewater discharge, where wastewater treatment plants only partially remove it.^8–10^ As a result, SMX has been found in up to 75% of European rivers studied, with concentrations averaging 76 ng L^–1^.^1^ Similar results have been reported in other regions, such as the Yongjiang River in China.^4^

To elucidate putative sources of SMX and to characterize environmental degradation, recent work has brought forward compound-specific stable isotope analysis (CSIA) as a powerful yet underutilized approach.^11–14^ CSIA provides valuable insight based on changes in the isotopic composition of the target compound at natural abundance by analyzing isotope ratios (e.g., ^13^C/^12^C and ^15^N/^14^N), rather than relying solely on concentration.^15–19^ The compounds’ isotope ratios are conventionally measured with a gas- or liquid-chromatography (GC or LC) hyphenated with isotope ratio mass spectroscopy (IRMS).^20–22^ After separation, the sample is converted into simple analyte gases (CO_2_ for ^13^C/^12^C, or N_2_ for ^15^N/^14^N), whose ratio is subsequently measured by IRMS. Subtle differences in these isotope ratios may serve for source fingerprinting, and changes in compound-specific isotope ratios can characterize reaction pathways when substances are transformed in natural processes. Recent studies specifically targeted the antibiotic SMX to explore its compound-specific isotope fractionation for degradation processes in natural and engineered systems, including photolysis,^11,12,14^ oxidation with ozone and chlorine dioxide,^23^ transformation by heat-activated persulfate,^24^ as well as microbial degradation.^11,13^ While most of these studies employed LC-IRMS for carbon isotope analysis,^11,12,23^ more recently, a method for derivatization has been developed, extending CSIA of SMX also to nitrogen isotope analysis.^14,25^

The information from conventional CSIA is limited, however. First, CSIA can analyze only one element at a time since the IRMS can detect only one gas species per measurement. This means that multiple injections in different instrument configurations are needed to acquire multielement isotopic information. Second, during degradation processes, changes in isotope ratios occur mainly at the reactive site.^26^ In contrast, isotope ratios measured by LC- and GC-IRMS represent the average across the entire compound. Hence, (i) isotope effects at specific reactive sites are diluted by contributions from nonreactive positions in the measurement of compound-average values, and (ii) the mechanistic information about the location of the reactive site gets lost.

In recent years, Orbitrap mass spectrometry (Orbitrap-MS) protocols have been advanced to overcome these limitations by fragment- and even position-specific isotope analysis of organic compounds.^27^ This represents a decisive advance also in comparison to nuclear magnetic resonance spectroscopy (NMR), which can deliver position-specific information but requires high amounts of pure analyte, and further compared to multicollector gas source mass spectrometry and GC-pyrolysis-GC-IRMS, which are restricted to small, specific analytes.^28–30^

With Orbitrap-MS, it is possible to simultaneously measure isotope ratios of various elements while retaining structural information. After ionization by either electron impact or electrospray ionization (ESI), the target analyte can be fragmented either directly after ionization, known as “source fragmentation”, or in the collision cell. The resultant fragments are subsequently analyzed in the Orbitrap mass analyzer, which provides isotope ratios of these fragments. In most cases, such fragment-specific values reflect multiple positions of the same element and are, therefore, not yet representative of individual molecular sites (i.e., position-specific values). Complementary information from different fragments with different positions can allow calculating the isotopic composition at each position by matrix algebra. For carbon isotope ratios, this approach, which calculates position-specific isotope values from a set of measured fragment-specific isotope values, was successfully applied to amino acids and gluconate.^31–34^ For sulfamethoxazole, three fragments have been identified: the aniline ring, the SO_2_ group, and the 3-amino-5-methyl isoxazole ring. After positive ionization and suitable fragmentation in the collision cell, two of these three fragments are formed, highlighting the potential for simultaneous carbon and nitrogen fragment-specific isotope analysis.^35^ Although no further fragments were obtained that would allow for calculation of position-specific isotope values, even such fragment-specific isotope values already give access to more dimensions of isotope information and, therefore, provide valuable information compared to the compound-average of CSIA.

However, isotope measurements by Orbitrap-MS can express differences only relative to a working standard. Although the same is true for GC-IRMS, the practice of quantitatively converting analytes into the same, universal measurement gases (CO_2_ for ^13^C/^12^C, or N_2_ for ^15^N/^14^N) makes it easier to introduce appropriate universal referencing schemes in GC-IRMS. To this end, organic reference materials for hydrogen, carbon, and nitrogen are available that are characterized against international reference materials, allowing for measurements to be linked to the respective international scales.^36,37^ In comparison, isotope ratios of organic compounds determined by Orbitrap-MS are highly dependent on the molecular structure of the respective compound. Therefore, a set of analyte-specific reference materials with known isotopic signatures at specific positions within the target compound is essential.

So far, standards for position- and fragment-specific Orbitrap isotope analysis have been characterized by either NMR or quantitative conversion of a functional group to CO_2_ for subsequent GC-IRMS measurement. A study on methionine took advantage of different methionine products characterized by NMR to generate a set of standards for Orbitrap-MS measurements.^31^ However, when moving to larger organic compounds, such as SMX, characterization by NMR becomes challenging, and to our knowledge, it is limited to carbon isotope analysis. Position-specific stable isotope analysis of alanine and serine relied instead on the virtue of the ninhydrin reaction, which converts the carboxy group of amino acids to CO_2_, allowing subsequent GC-IRMS measurements and informing about the isotope values specifically in this position.^32,33^ For alanine, the remaining acetaldehyde was further separated on a GC-pyrolysis-GC-IRMS, resulting in the isotope value of an additional position.^33^ While elegant, this specific approach for amino acids is not applicable to a more diverse set of compounds. Finally, mixed results are reported from attempts to calibrate functional group-specific isotope ratios (here: PO_3_^–^) when derived from different analytes (phosphate vs aminophosphonates) through fragmentation. While this approach was successful for (2-aminoethyl)­phosphonic acid, it did not succeed for aminomethyl phosphonic acid.^38^

To lay the foundation for fragment-specific stable isotope analysis of SMX, it was therefore our objective to prepare reference materials with ^13^C and ^15^N enrichment at specific positions of SMX. Since such singly labeled SMX is not commercially available, we aimed (i) to develop a synthesis strategy by adapting known synthesis routes, starting with simple building blocks that are available with ^13^C and ^15^N labels, respectively. In the synthesis route, it was crucial to avoid any synthetic steps that may cause dilution or scrambling of the isotopic label, requiring regioselective reactions. (ii) Second, it was our objective to mix these labeled synthesis products with SMX at natural abundance to achieve isotopic homogeneity at the 0.4 mg level. (iii) Further, we aimed to demonstrate successful label incorporation at the EA-IRMS bulk level and to confirm with Orbitrap-MS that the label was specifically introduced into the intended fragments. (iv) Subsequently, it was our goal to use these standards for calibration of the Orbitrap-MS for isotope measurements of SMX. (v) At last, we aimed to deliver a proof-of-principle for the use of these materials as standards to measure fragment-specific changes in carbon and nitrogen isotope ratios during a model transformation reaction of SMX.

## Experimental Section

### Chemicals and Stock Solutions

Sulfamethoxazole (analytical standard, BCCH8532 for standard preparation, SMX_0_), acetonitrile (“HPLC-grade”), and methanol (as Orbitrap eluent: “hypergrade for LC–MS”; as HPLC eluent: “HPLC-grade”) were purchased from Sigma-Aldrich (Germany); formic acid (ROTIPURAN ≥ 99.0%, LC–MS grade) was from Carl Roth (Germany). The used water (18.2 MΩ cm at 25 °C) was from a Milli-Q Reference water purification system (Merck Millipore, USA). All standard solutions of SMX for ESI-Orbitrap-MS measurements were stored at 4 °C until further use. A summary of the used ^13^C-labeled materials is provided in Table 1.

**Table 1:** Labeled Starting Materials

compound	source	label	supplier
acetanilid-^15^N	MBBD8841	99 atom % ^15^N	Sigma-Aldrich
acetanilid-^15^N	MBBC9911	99 atom % ^15^N	Sigma-Aldrich
acetonitrile-^15^N	MBBD4883	99 atom % ^15^N	Sigma-Aldrich
ethyl acetate-1-^13^C	MBBD7948	99 atom % ^13^C	Sigma-Aldrich

### Total Synthesis of ^13^C- and ^15^N-Labeled Sulfamethoxazole

Air- and moisture-sensitive reactions were carried out in oven-dried glassware using the standard Schlenk techniques with dry argon as the inert gas. Air-stable solids were added against the flow of argon. Liquids and solutions were transferred using a syringe with a cannula. A balloon filled with argon maintained positive argon pressure during the performed reactions. In the synthesis procedure, amounts used refer to those without a stable isotope label inside the starting material. For reactions with labeled starting materials, the amounts were adjusted accordingly. Depending on the position of the label, some nonlabeled building blocks were purchased instead of being synthesized according to the procedure. A detailed summary of the chemicals and solvents used (Section S1.1), besides the labeled starting materials (Table 1), chromatography (Section S1.2), nuclear magnetic resonance spectroscopy (Section S1.3), and characterization of all synthesis steps (Sections S1.4 and S1.5) is included in Supporting Information S1.

#### 3-Hyroxybutyronitrile (**1a-c**)

Based on the procedure of Boers et al.,^39^ to a solution of diisopropylamine (53.2 mmol, 1.06 equiv) in THF (120 mL), *n*-butyllithium (52.8 mmol, 1.6 M in hexanes, 1.05 equiv) was added dropwise at −78 °C. After removing the cold bath for 10 min, the reaction was cooled to −78 °C, acetonitrile (51.7 mmol, 1.00 equiv) in THF (10 mL) was added dropwise, followed by the addition of ethyl acetate (51.7 mmol, 1.00 equiv) in THF (10 mL) 15 min later. The reaction mixture was stirred at −78 °C for 5 h, NaBH_4_ (31.7 mmol, 0.64 equiv) was added, the cold bath was removed, and the reaction mixture was stirred overnight. Under vigorous stirring at 0 °C, HCl (6 M, 20 mL) was added. Subsequently, the reaction mixture was heated to 55 °C for 15 min. After decanting the solution, the residue was washed twice with ether. The combined organic phase was washed with a saturated NaCl solution. The aqueous layer was extracted with ether, and the combined organic phase was dried over MgSO_4_. After removal of the solvent under reduced pressure, the crude product was purified by flash column chromatography with ether as eluent, yielding 3-hydroxybutyronitrile (**1a**–**c**) as a slightly yellow to colorless oil.

#### Crotononitrile (**2a-c**)

To a suspension of 3-hydroxybutyronitrile (**1a**–**c**) (15.1 mmol, 1.00 equiv) in Ph_2_O (2.3 mL), Na_2_B_4_O_7_ (6.5 mmol, 0.43 equiv) was added, and the reaction was heated to 190 °C for 48 h, while the desired reaction product was continuously removed by distillation. Separation of the product from the aqueous layer resulted in crotononitrile (**2a**–**c**) as a slightly yellow oil.

#### 3-Amino-5-methyl-isoxazole (**3a-c**)

According to Klötzer et al.,^40^ to a solution of crotononitrile (**2a**–**c**) (28.1 mmol, 1.00 equiv) in MeOH (2.3 mL), Br_2_ (56.2 mmol, 2.00 equiv) was added dropwise. The reaction was stirred at 0 °C overnight, then continued at room temperature for 24 h in the dark. Next, NaOH (15 wt %, 7.7 mL) was added to the reaction solution at 0 °C. The solution received was added to a solution of hydroxyurea (28.5 mmol, 1.02 equiv) in NaOH (10 wt %, 23 mL) and vigorously stirred for 72 h at room temperature. After stirring for 2 h at 50 °C, the reaction solution was extracted with ethyl acetate. The solvent of the combined organic layers was removed under reduced pressure, yielding 3-amino-5-methyl-isoxazole (**3a**–**c**) as a light brown solid.

#### 4-Acetamidobenzene-1-sulfonyl Chloride (**4a-b**)

According to Zhang et al.,^41^ while stirring acetanilid (16.9 mmol, 1.00 equiv) at 0 °C, chlorosulfonic acid (88.1 mmol, 5.21 equiv) was added dropwise. The purple reaction solution was stirred for 1 h at 0 °C and 2 h at 70 °C. The reaction solution was cooled to room temperature and slowly poured onto crushed ice. The formed precipitate was filtered, washed with ice-cold water, and dried under vacuum overnight, resulting in 4-acetamidobenzene-1-sulfonyl chloride (**4a**–**b**) as a white solid.

#### Sulfamethoxazole (**5a-d**)

Based on Brimacombe et al.,^42^ to a solution of 3-amino-5-methyl-isoxazole (**3a**–**c**) (13.8 mmol, 1.15 equiv) in CH_2_Cl_2_ (20 mL), 4-acetamidobenzene-1-sulfonyl chloride (**4a**–**b**) (12.0 mmol, 1.00 equiv) and pyridine (49.6 mmol, 4.13 equiv) were added. The reaction mixture was stirred at room temperature overnight. The solvent was removed under reduced pressure, NaOH (20 wt %, 20 mL) was added, and the resulting reaction solution was stirred at 100 °C for 3 h. The reaction solution was cooled to room temperature and extracted with EtOAc (30 mL). The residual aqueous layer was suspended in EtOAc (250 mL), and, under vigorous stirring, HCl (2 M) was dropwise added to a pH of 5. Subsequently, the two layers were separated, the organic layer was dried over MgSO_4_, and the solvent was removed under reduced pressure. The resulting sulfamethoxazole (**5a**–**d**) was obtained as an off-white solid.

### Standard Preparation

High purity reference compounds for Orbitrap-MS are required for both direct infusion Orbitrap-MS measurements and isotope measurements by EA-IRMS. The commercially available nonlabeled sulfamethoxazole used as a starting material appeared as a white solid, whereas the self-synthesized sulfamethoxazole exhibited an off-white color. This discoloration indicated traces of byproducts from the synthesis process. Such impurities could potentially cause biased isotope values in the resulting standard. Therefore, the required quantities for standard preparation of each respective ^13^C- or ^15^N-labeled SMX were purified by reversed-phase column chromatography using acetonitrile and water as mobile phase (Section S2). After collecting the target fractions, the solvent was removed under reduced pressure.

When preparing isotope standards by mixing labeled and nonlabeled compounds, the resulting powder must be homogeneous so that every subsample has the same isotopic composition, ensuring measurements are consistent and comparable. Therefore, labeled SMX was dissolved in 2.5 mL acetonitrile, achieving an estimated concentration of ∼2 g L^–1^ for ^13^C_1_–SMX (**5c**) and 0.7 g L^–1^ for ^15^N_1_–SMX (**5b**) and (**5d**). This stock solution was distributed into 50 mL round-bottom flasks at target enrichments of 50‰ (250 μL), 100‰ (500 μL), and 200‰ (1000 μL), and the solvent was removed under reduced pressure. Next, 1 g of SMX_0_ was added, followed by the addition of 3 mL of acetonitrile. The mixture was then heated to 70 °C to ensure complete dissolution of both the labeled and nonlabeled SMX. If the SMX did not completely dissolve at this temperature, additional acetonitrile was added until complete dissolution was achieved. Afterward, the hot solution was rapidly poured into a 250 mL round-bottom flask containing 100 mL of vigorously stirred cold water, resulting in a rapid precipitation of SMX. While transferring the hot SMX-acetonitrile solution, it was ensured that no precipitation occurred before pouring the solution into the water. In the next step, the aqueous phase was removed from the precipitate. To minimize the loss of precipitate and prevent contamination, the aqueous supernatant was removed by sucking through a Teflon cannula (Ø 0.3 mm) equipped with a self-made filter frit (glass microfiber filters, GF/C, Ø 24 mm, Whatman, GE Healthcare Life Sciences), secured in place with PTFE sealing tape (W 1/2 in., Sigma-Aldrich) driven by application of vacuum. All precipitates were dried overnight under vacuum, resulting in white powders, which were stored at 4 °C in the dark in amber glass vials. These standards were characterized by EA-IRMS (Section S3) and ESI-Orbitrap-MS (see below).

### Transformation Reaction of Sulfamethoxazole

Reductive transformation of sulfamethoxazole by Fe­(II) and goethite performed according to Mohatt et al.^43^ is described in detail in Section S4, including chemicals and methods used (Section S4.1), the degradation experiment (Section S4.2), extraction of the sample using solid-phase extraction (Section S4.3), and GC-IRMS measurements (Section S4.4).

### Orbitrap Instrumentation

A Vanquish liquid chromatography system was coupled with an ESI Orbitrap Exploris 240 mass spectrometer (both, Thermo Fisher Scientific, Germany). The Vanquish liquid chromatography system consisted of a Split Sampler HT VH-A10-A, Binary Pump N VN-P10-A (“low-flow pump”), Binary Pump H VH-P10-A (“high-flow pump”), Diode Array Detector (DAD) FG VF-D11-A, and Column Compartment VH-C10-A (all, Thermo Fisher Scientific, Germany). The sample was introduced into the electrospray ionization (ESI) source in two ways: either by the low-flow pump via the autosampler of the Vanquish system (total loop volume: 130 μL, Thermo Fisher Scientific, Germany) or through a syringe pump (KDS-230-CE, kdScientific, USA) at a flow rate of 4 μL min^–1^. For ionization, the atmospheric pressure ion source OptaMax NG was used with a nonheated ESI probe equipped with a low-flow needle insert (Thermo Fisher Scientific, Germany).

The positive ionization mode was selected for analyzing the two target fragments. The ESI spray was optimized before each sequence to ensure a stable and intense TIC (relative standard deviation < 8%) by adjusting the spray voltage, sheath gas (30), and auxiliary gas values (0). After ionization, the continuous beam of ions was filtered by an advanced quadrupole technology (AQT) mass filter, which, in our case, allowed only ions with a *m*/*z* between 251.5 and 256.5 to pass. They were subsequently transmitted through the curved linear trap (C-trap) and stored as packages in the ion-routing multipole (IRM), with their amount controlled by the automatic gain control (AGC) algorithm (here: 1.0 × 10^6^, 1.5 × 10^6^, and 3.0 × 10^6^). Fragmentation of the ionized molecular ion, *m*/*z* 254, was induced by higher energy collisional dissociation (HCD) in the IRM. The accumulated fragment ions were transferred back through the C-trap into the Orbitrap mass analyzer for measurement. The oscillation of each ion package within the analyzer was recorded as image current and stored as a transient. By adjusting the microscan setting, 10 transients were averaged before performing an enhanced Fourier transform (eFT) to generate the mass spectrum. The instrument parameters used and the analyzed fragments are summarized in Table 2 and Scheme 1, respectively.

**Table 2:** Instrument Settings Used for the Fragment-Specific Isotope Analysis of SMX

	F92 & F99
polarity (±)	positive
ion transfer tube temperature (°C)	320
nominal orbitrap resolution	90,000
AQT isolation window (*m*/*z*)	251.5–256.5
absolute AGC target (#)	1.0 × 10^6^, 1.5 × 10^6^, or 3.0 × 10^6^ ^a^
microscans (#)	10
RF lens (%)	100
maximum ion injection time (ms)	1000
precursor ion (*m*/*z*)	254
absolute HCD collision energy (V)	20

a 3.0 × 10^6^ was selected for further measurements.

**Scheme 1:**
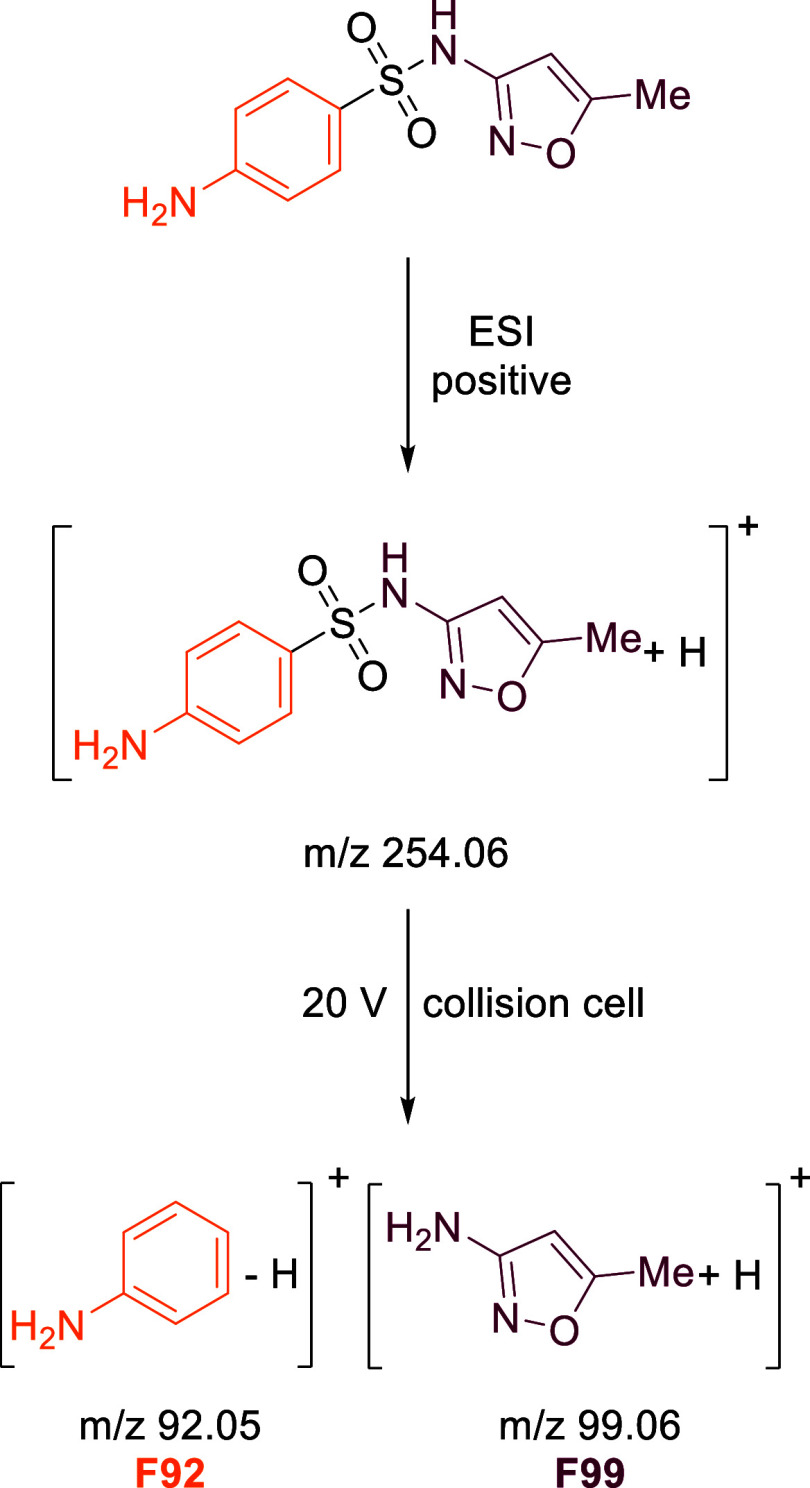
Fragmentation of Sulfamethoxazole after Positive Electrospray Ionization and Fragmentation by HCD in the IRM

#### Standard Measurements and the Effect of the AGC Target

For experiments calibrating the Orbitrap-MS for isotope analysis, the autosampler was directly connected to the Orbitrap-MS without a separation column, using the low-flow pump under isocratic conditions (36% MeOH in H_2_O with 0.1% formic acid). For each injection, 80 μL of the respective SMX solution was injected at a flow rate of 4 μL min^–1^, resulting in ∼20 min of useable data for isotopologue ratio analysis. To ensure the absence of carryover, the flow rate was increased after 24 min to 20 μL min^–1^ and switched back to 4 μL min^–1^ after 29.9 min. Standards with different enrichments at the respective positions were injected and bracketed with injections of SMX at natural abundance (SMX_0_), which served as a reference. Each standard was prepared as a 5 μM solution with 36% MeOH in H_2_O with 0.1% formic acid and was measured in quintuplicate. All standards were measured using an AGC target of 1.0 × 10^6^, 1.5 × 10^6^, and 3.0 × 10^6^, noting that for further measurements, an AGC target of 3.0 × 10^6^ was used.

#### Determination of Isotope Values of Samples

An online LC-ESI-Orbitrap-MS system was used.^35^ Briefly, 40 μL of an SMX solution (100 μM) were injected. The chromatographic separation of SMX was performed on a C18 column (250 × 4.6 mm, Luna 5 μm C18(2) 100 Å, Phenomenex) with a flow rate of 0.5 mL min^–1^ under isocratic conditions (36% MeOH in H_2_O with 0.1% formic acid). The chromatographic peak of SMX was captured and homogenized in a dynamic mixing chamber (V7119-1, volume 1140 μL, Knauer, Germany) and continuously delivered into the Orbitrap-MS at a flow rate of 4 μL min^–1^ under isocratic conditions (36% MeOH in H_2_O with 0.1% formic acid). The isolated SMX was bracketed with a 6 μM SMX solution from the syringe pump. This led to five syringe and four sample blocks, each with a duration of 18 min.

### Data Processing

For all Orbitrap experiments, a 15 min time interval was used for data processing. Ion intensities and noise for selected isotopic peaks were recovered from instrument.RAW files using IsoX (Thermo Fisher Scientific). The number of ions observed (IO) per scan *N*_IO_ was calculated by eq 1.^27^

1 $$N_{IO}=\frac{S}{N}\cdot \frac{C_{N}}{z}\cdot {\left(\frac{R_{N}}{R}\right)}^{1/2}\cdot \mu^{1/2}$$

here, *S* is the ion intensity, *N* is the peak noise, *C*_N_ is an empirical constant measured by Makarov and Denisov^44^ relating the signal-to-noise ratio to ion counts (here: 3), *z* is the charge (here: 1), *R*_N_ is the reference resolution of 240,000, *R* is the nominal mass resolution, and μ is the number of microscans (here: 10). We extracted the peaks of the following ions: ^12^C_6_^1^H_6_^14^N (*m*/*z* 92.0493), ^12^C_6_^1^H_6_^15^N (*m*/*z* 93.0465), and ^13^C_1_^12^C_5_^1^H_6_^14^N (*m*/*z* 93.0532) for F92, ^12^C_4_^1^H_7_^14^N_2_^16^O (*m*/*z* 99.0552), ^12^C_4_^1^H_7_^14^N^15^N^16^O (*m*/*z* 100.0525), and ^13^C_1_^12^C_3_^1^H_7_^14^N_2_^16^O (*m*/*z* 100.0587) for F99. The isotopologue ratios *R* ^*i*^ were calculated as the ratio of the summed number of ions observed *N*_IO_ of isotopologue *i* over the measurement time to those of the basepeak, according to Hilkert et al.^45^ (eq 2).

2 $$R^{i}=\frac{\sum_{j=1}^{N}N_{IO}\;\left(isotopologue\;i\right)}{\sum_{j=1}^{N}N_{IO}\;\left(basepeak\right)}$$

For comparison with IRMS, we assumed a stochastic distribution of multiply substituted isotopologues within the analyzed fragment ion. This assumption enables the interpretation of isotopologue ratios obtained from Orbitrap-MS as corresponding to the isotope ratios. For example, the ratio of the ^13^C-substituted F99 ion to the unsubstituted F99 ion can be understood as representing the ^13^C/^12^C isotope ratio. Isotope ratios derived from EA-IRMS measurements are reported in the δ-notation in per mil (‰) relative to the international reference material Vienna PeeDee Belemnite (VPDB) for δ^13^C and air for δ^15^N using eq 3.

3 $$\delta^{h}E=\frac{R_{sample}-R_{reference}}{R_{reference}}$$

here, δ^h^ *E* is the δ-value of a given element, *R*_sample_ is the isotope ratio of the sample, and *R*_reference_ is the isotope ratio of the reference of the given element *E*. Shifts of isotopic signatures are reported as the deviation Δδ^h^ *E*, between the isotope ratios in the sample (δ^h^ *E*_sample_) and a reference (δ^h^ *E*_reference_), according to eq 4.

4 $$\Delta \delta^{h}E=\delta^{h}E_{sample}-\delta^{h}E_{reference}$$

For the standard characterization experiments, using direct infusion from the autosampler, the average isotope ratio of the 5 μM SMX_0_ bracketing solution applied before and after each standard (i.e., SMX A1) measurement was used as the *R*_reference_, with its δ^h^ *E*_reference_ value arbitrarily set to 0‰, expressing isotope ratios as deviations from SMX_0_. For the LC-ESI-Orbitrap-MS setup, each captured and homogenized standard was bracketed with 6 μM SMX from the syringe. The syringe bracketing solutions averaged from before and after the sample measurement were used as *R*_reference_. Here, we used a switching time of 3 min, meaning that the first 3 min of each block were not used for data evaluation. The δ^h^ *E* value of SMX_0_ was set to 0, and the other standards are reported against this value. A two-point calibration was used to calibrate sample isotope values using the predetermined isotope values of the reference standards. It is important to note that we adhered to the principle of identical treatment for both the reference and the sample. This means that instead of using direct syringe infusion as our reference for SMX_0_, we also processed the standard through the column. All reported errors represent the 95% confidence intervals (CIs). Therefore, the error of the average ratio from the bracketing standard SMX_0_ or syringe infusion measured before (Std1) and after (Std2) the different standard or sample was estimated by eq 5 using the standard error of the mean of each respective ratio.

5 $$\sigma_{reference-average}=\sqrt{{\left(\frac{\sigma_{Std1}}{2}\right)}^{2}+{\left(\frac{\sigma_{Std2}}{2}\right)}^{2}}$$

The standard error of the mean of *R*_sample_, σ_sample_, and σ_reference_ were propagated using the Gaussian error propagation (eq 6) and are stated in ‰.

$$\sigma_{\delta ‐value}=\sqrt{{\left(\frac{\sigma_{sample}}{R_{reference-average}}\right)}^{2}+{\left(\frac{R_{sample}\cdot \sigma_{reference-average}}{R_{reference-average}^{2}}\right)}^{2}}$$ 6

Such errors were obtained for each reference sample bracketing and further propagated by eq 7, resulting in 95% CI

7 $$\sigma_{95\%\;CI}=\left(\frac{1}{n}\cdot \sqrt{\sum_{i=1}^{n}\sigma_{i}^{2}}\right)\cdot t_{\alpha ,n-1}$$

here, *t*_α,*n*–1_ is the Student *t*-factor. For our standard characterization experiments, we used a value of 2.776 (*n* = 5); for the sample measurements with the chromatography system, we used a Student *t*-factor of 3.182 (*n* = 4).

## Results and Discussion

### Synthesis of ^13^C- and ^15^N-Labeled Sulfamethoxazole

Scheme 2 illustrates our synthetic strategy for preparing SMX with intramolecular ^13^C and ^15^N labels. We successfully synthesized SMX starting with ethyl acetate and acetonitrile, which are simple molecules commercially available with ^13^C or ^15^N labels, respectively (pink and purple labels in Scheme 2). Three steps were required to form the five-membered heterocycles **3b** and **3c**.^39,40^ The final step involved the formation of the sulfonamide through a reaction between **3b** or **3c** and acetamidobenzene-1-sulfonyl chloride (**4a**).^42^ The yields over the four steps for the ^13^C label within the isoxazole ring or the ^15^N label at the sulfonamide group were 13% (**5c**) and 8% (**5d**), respectively. On the other hand, SMX with a ^15^N label at its amine group required only two steps (orange labels in Scheme 2), chlorosulfonation of ^15^N-labeled acetanilide with chlorosulfonic acid, followed by the sulfonamide formation.^41^ The overall yield for **5b** was 32%. Overall, the synthesis performed with the expensive stable isotope-labeled precursors gave a modest yield (average yields per step >50%); however, only small quantities are required for standard preparation (mixing 1 g of nonlabeled SMX with 1 mg of ^13^C-labeled SMX results in an enrichment of ∼100‰ at the specific position), and extra material was prepared for future studies. The ^13^C and ^15^N labels were successfully confirmed by NMR spectroscopy and tracked throughout the entire synthesis procedure (Sections S1.4 and S1.5).

**Scheme 2:**
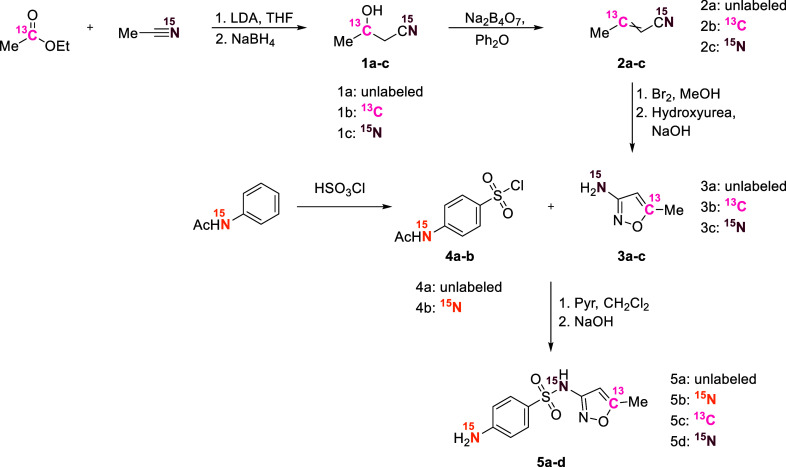
Overview of the Reactions Used for ^13^C- and ^15^N-Labeled SMX

### Preparation of SMX Standards with Homogeneity at the 0.4 mg Level

For the generation of reference standards, isotopic homogeneity is mandatory, since any aliquot withdrawn from the stock of reference material must have a constant, defined isotope ratio. Consequently, solid reference material must be precipitated instantaneously. This ensures that isotope effects during crystallization—which would enrich isotopes in the solid relative to the liquid phase and lead to different isotope values of “early” vs “late” precipitates—are avoided.^37^ Traditional methods for homogenization include (i) shock-freezing by dripping aqueous solutions of labeled and nonlabeled compounds into liquid nitrogen, followed by freeze-drying, or (ii) rapid cooling of a homogeneous melt, resulting in instantaneous crystallization.^36^ However, SMX is only slightly soluble in water < 1 L^–1^ and decomposes shortly above its melting point, making these literature procedures unsuitable. Therefore, we developed a new procedure by dissolving labeled and nonlabeled SMX in as little hot acetonitrile as possible and pouring the mixture into ice-cold water, resulting in instantaneous precipitation. After removing the aqueous phase and drying the precipitate under vacuum, EA-IRMS measurements demonstrated that the prepared standards were homogeneous at the 0.4 mg level (*n* = 5, standard deviation < 0.3‰).

### Confirmation of Position-Specific Label Introduction in the Prepared Standards

The fragmentation pattern of SMX in the HCD cell after positive ionization resulted in two key fragments: F92, representing the aniline ring, and F99, representing the isoxazole ring (Scheme 1). The aniline moiety of the molecule contains only one nitrogen atom; therefore, in this case, the term position-specific isotope analysis can be used. However, this is not possible for all carbon atoms or the two nitrogen atoms within the heterocycle. Therefore, we will continue using the term fragment-specific isotope analysis for all isotopologues of these two fragments. For the two target fragments, we prepared isotope standards using the synthesized labeled SMX (**5b**–**d**). To this end, SMX at natural abundance (SMX_0_) was fortified with small amounts of the respective position-enriched SMX (**5b**–**d**) and homogenized with our developed procedure, resulting in the respective standards A-D 1, 2, and 3 (Figure 1). These standards were subsequently analyzed for their molecular average and fragment-specific carbon and nitrogen isotope values using EA-IRMS and ESI-Orbitrap-MS, respectively. Bulk isotope values from EA-IRMS confirmed that greater spikes of labeled SMX resulted in more positive isotope values of the respective standards (Table S2). These bulk isotope values subsequently served to calculate the enrichment in F92 and F99, Δδ^13^C_calc_ and Δδ^15^N_calc_, which are reported relative to the molecular average of SMX_0_. Figure 1 shows that the fragment-specific isotope data by ESI-Orbitrap-MS only showed increases in the Δδ^13^C and Δδ^15^N values at the position that had been labeled, confirming that no reshuffling of isotopes occurred during analysis with ESI-Orbitrap-MS.

**Figure 1:**
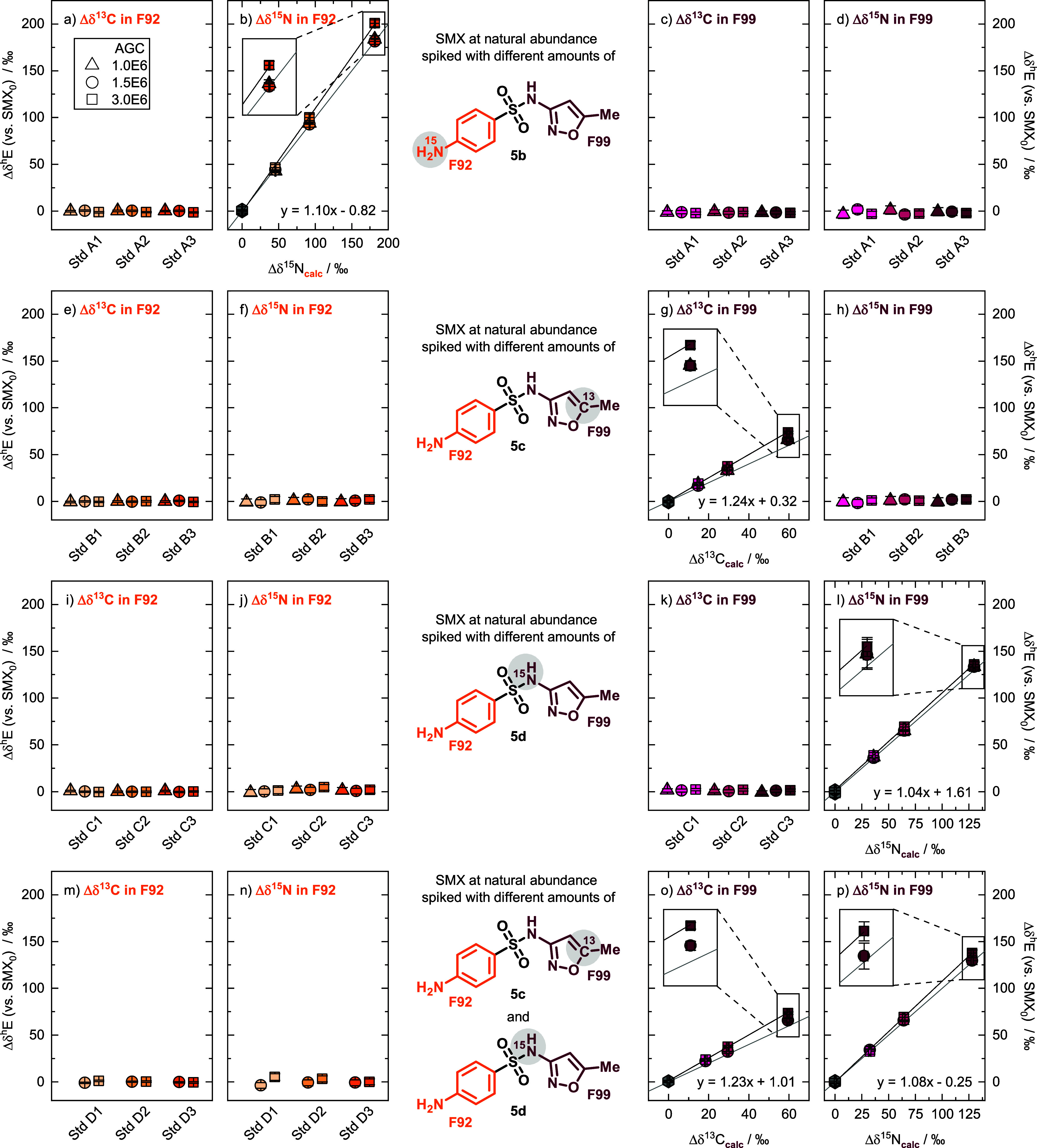
Standards A1–3 (a–d), B1–3 (e–h), C1–3 (i–l), and D1–3 (m–p) measured on the Orbitrap-MS by direct infusion showing Δδ^13^C and Δδ^15^N values of both fragments F92 and F99 against SMX_0_ using different AGC targets, displaying the fit of the linear regression for the calibrations at an AGC target of 3.0 × 10^6^ (black line) and the 1:1 lines (gray line); the error bars represent 95% CIs. Note that different measurement days resulted in different slopes for the same fragment (e.g., Δδ^15^N in F99).

### Calibration for Fragment-Specific Carbon and Nitrogen Isotope Measurements

We successfully calibrated our ESI-Orbitrap-MS method using our prepared standards for fragmentation-specific nitrogen stable isotope analysis of F92 and F99, as well as for fragmentation-specific carbon stable isotope analysis of F99 (Figure 1). All standards were measured using different AGC targets (1.0 × 10^6^, 1.5 × 10^6^, and 3.0 × 10^6^) while keeping the other instrument parameters constant. Since the best sensitivity was accomplished at an AGC target of 3.0 × 10^6^, we conducted all further measurements with this setting, resulting in 95% CIs for Δδ^13^C and Δδ^15^N in F92 of 0.7‰ and 3.4‰, respectively, and for Δδ^13^C and Δδ^15^N in F92 of 1.3‰ and 2.9‰, respectively. Figure 1 illustrates that our fragment-specific reference standards can calibrate slopes that deviate from unity. The slope deviating from unity can arise from (i) the calculation of the fragment-specific isotope values and (ii) the instrument causing isotope fractionation. (i) For each standard, the enrichment at the labeled position was calculated using the EA-IRMS-derived isotope values, while the isotope values of the nonenriched positions in the target fragment were assumed to follow a stochastic distribution and were therefore set equal to the overall bulk isotope value of SMX_0_. Therefore, intramolecular variation in SMX_0_ can contribute to slopes deviating from unity (scale compression), and we only report Δδ^h^ *E* values. (ii) Additionally, instrumental isotope fractionation causes slopes deviating from unity.^27^ For example, the AGC target set affected the measured carbon isotope values of F99 (Figure 1g), while no significant changes were observed in the nitrogen isotope values of F99 (Figure 1l). Taken together, within our study, we found reproducible calibration lines; the calibration line generated from the set of standards D (Figure 1o–p) can be successfully applied to the isotope measurements of the sets of standards B (Figure 1g) and C (Figure 1l), which were measured in different measurement sequences. Therefore, our synthesis strategy resulted in the successful preparation of fragment-specific standards for calibrating the ESI-Orbitrap-MS. For the subsequent quantification of changes in isotope values during the transformation reactions of SMX, we used only standard A3 and D3 for two-point calibration of each target fragment.

### Degradation of Sulfamethoxazole with Fe­(II)/Goethite

After 2 h of reductive transformation of SMX with Fe­(II) and goethite, we observed changes in the carbon and nitrogen isotope values of fragment F99 and considered changes greater than twice the 95% CIs as significant, e.g., 8‰ for nitrogen isotope values in F99. The carbon isotope ratio increased from −3 ± 2‰ to 4 ± 2‰, and the nitrogen isotope ratio from 2 ± 4‰ to 27 ± 4‰, while we did not observe any significant changes in the isotope ratios of either carbon or nitrogen in F92 (Figure 2). This agrees with the proposed reaction mechanism of this transformation process by Mohatt et al.,^43^ as the reaction pathway is initiated by the cleavage of an N–O bond in the isoxazole ring (Figure 3a), leading to transformation products that only change 3-amino-5-methyl-isoxazole (F99), while retaining the sulfanilamide part (F92). In Figure 3, we compared our data with conventional GC-IRMS measurements. While the Δδ^13^C of the GC-IRMS only showed a change of 2.5 ± 0.7‰, the change of Δδ^13^C in F99 was 7 ± 2‰ (Figure 3b). The compound’s average nitrogen isotope values showed a change of 12 ± 1‰, while the change in the Δδ^15^N was 26 ± 5‰ for F99 (Figure 3c). When converting the changes from the compound’s average to only the reactive fragment, this agrees with our Orbitrap-derived value for Δδ^13^C, while for Δδ^15^N we see a larger deviation which is likely attributable to the lower measurement precision of nitrogen isotope analysis compared to carbon. Hence the seeming discrepancy between the values to the right of Figure 3c (column “Orbitrap F99” vs “GC-IRMS 3/2”) rather reflects analytical uncertainty than mechanistic differences. Nonetheless, while GC-IRMS only provides the compound’s average, the isotope ratios determined by Orbitrap can provide simultaneous information on multiple elements and parts of the molecule, as illustrated by the values for “Orbitrap F92” and “Orbitrap F99” in Figure 3.

**Figure 2:**
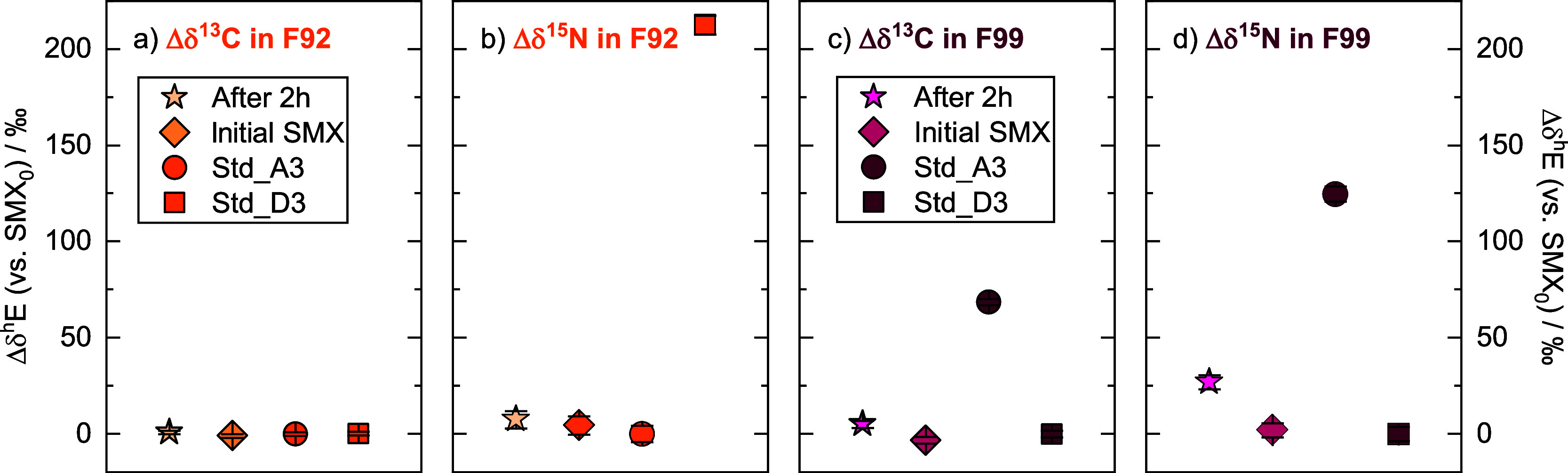
Measurement sequence on the ESI-Orbitrap-MS for stable (a + c) carbon and (b + d) nitrogen isotope analysis of F92 and F99 for two samples, followed by two standards for calibration.

**Figure 3:**
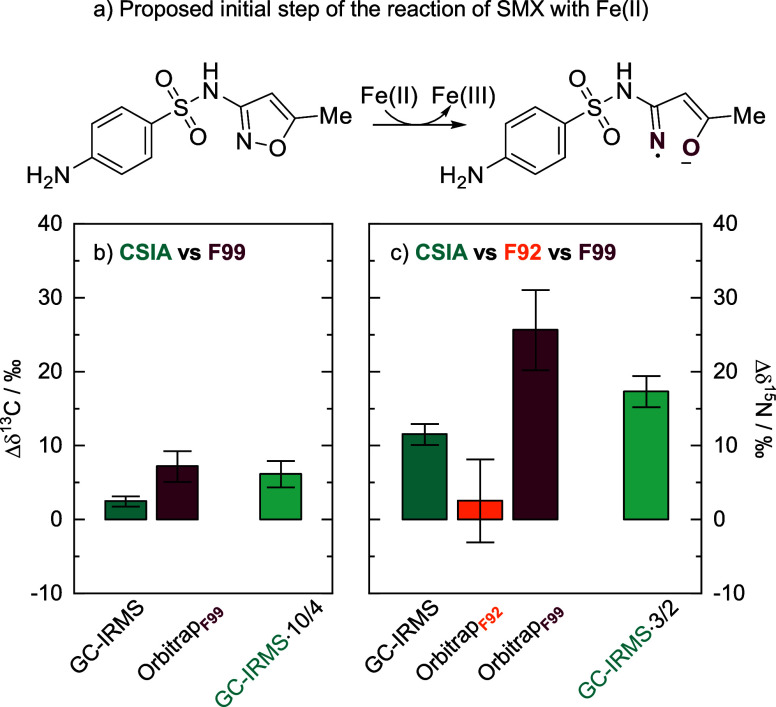
(a) Proposed initial step of the reductive transformation of SMX by Fe­(II) and goethite,^43^ and the associated changes in (b) carbon and (c) nitrogen values, comparing compound-specific stable isotope analysis by GC-IRMS with fragment-specific stable isotope analysis by ESI-Orbitrap-MS; the error bars represent propagated 95% CIs.

## Conclusions

In this work, we demonstrate the use of ESI-Orbitrap-MS for the fragment-specific isotope analysis of carbon and nitrogen in two fragments of SMX simultaneously. Using position ^13^C- and ^15^N-labeled standards with known bulk isotope values, we showed that the ESI-Orbitrap-MS method reliably detects isotopic enrichment in the respective fragments, where uncertainties in isotope values were about three times greater for nitrogen than for carbon. The position-enriched standards proved essential for method development, verifying the absence of recombination during ionization, and calibrating Orbitrap-MS against EA-IRMS values. Our developed method, combined with online hyphenation of LC with ESI-Orbitrap-MS, highlights the potential to serve as a complementary approach to conventional LC- and GC-IRMS measurements in SMX degradation studies, providing additional insights into degradation pathways based on fragment-specific isotope information at natural abundance.^35^

## Supplementary Material



## Data Availability

The ESI-Orbitrap raw data supporting the findings of this study are available at http://10.5281/zenodo.17671343.

## Notes

Further details on the total synthesis of labeled sulfamethoxazole, including chemicals and solvents used, chromatography, nuclear magnetic resonance spectroscopy, characterization of the synthesis products, and ^1^H- and ^13^C NMR spectra; reversed-phase liquid chromatography procedure; EA-IRMS procedure and results; transformation of sulfamethoxazole with chemicals and methods used, transformation experiments, solid-phase extraction of sulfamethoxazole, and GC-IRMS procedure; and concentration analysis by HPLC (PDF)
