# Supplementary material for: Intramolecularly Labeled Reference Standards of Sulfamethoxazole for Fragment-Specific Isotope Analysis by Electrospray Ionization Orbitrap Mass Spectrometry
Source: J Am Soc Mass Spectrom. 2026 Feb 6;37(3):717–26. doi: 10.1021/jasms.5c00402 (PMC12964526; doi:10.1021/jasms.5c00402)
Supplement: Supplementary file 1 [file js5c00402_si_001.pdf]

Supporting Information for the *Journal of the American Society for Mass Spectrometry*

Intramolecularly Labeled Reference Standards of Sulfamethoxazole for Fragment-Specific Isotope Analysis by Electrospray Ionization Orbitrap Mass Spectrometry

Aoife Canavan[a], Christopher Dirr[a], Martin Elsner[a,*]

[a]Technical University of Munich, TUM School of Natural Sciences, Department of Chemistry, Chair of Analytical Chemistry and Water Chemistry, Lichtenbergstraße 4, 85748 Garching, Germany

*Corresponding author: Martin Elsner
Address: Lichtenbergstraße 4, 85748 Garching, Germany
Phone: +49 (89) 289 - 54500
E-mail: m.elsner@tum.de

Contents

S1 Total Synthesis of ^{13}C- and ^{15}N-Labeled Sulfamethoxazole S-2
S1.1 Chemicals and Solvents S-2
S1.2 Chromatography S-2
S1.3 Nuclear Magnetic Resonance Spectroscopy S-2
S1.4 Characterization of Synthesis Products S-3
S1.5 ^{1}H- and ^{13}C-NMR Spectra S-7
S2 Reversed-Phase Liquid Chromatography S-16
S3 Elemental Analysis Isotope Ratio Mass Spectrometry S-16
S4 Reductive Transformation of Sulfamethoxazole S-17
S4.1 Chemicals and Methods S-17
S4.2 Transformation of Sulfamethoxazole using Fe(II) and Goethite S-17
S4.3 Solid-Phase Extraction of Sulfamethoxazole S-18
S4.4 GC-IRMS Measurements S-18
S5 Concentration Analysis S-20
S6 References S-20

S1 Total Synthesis of ^{13}C- and ^{15}N-Labeled Sulfamethoxazole

S1.1 Chemicals and Solvents

The used water (18.2 MΩ cm at 25 °C) was from a Milli-Q® Reference water purification system (Merck Millipore, USA). Acetanilid (99%), acetonitrile (anhydrous, 99%), 3-amino-5-methyl-isoxazole (≥97%), chlorosulfonic acid (99%), diisopropylamine (99.95%), ethyl acetate (anhydrous, ≥99.7%), hydrochloric acid (puriss., p.a., ACS reagent, ≥37%), magnesium sulfate (puriss. p.a., drying agent, anhydrous, ≥98.0%), N-acetylsulfanilyl chloride (98%), n-butyllithium (1.6 M in hexanes), pyridine (anhydrous, 99.8%), sodium borohydride (≥98.0%), sodium chloride (ACS reagent, ≥99.0%), sodium tetraborate (anhydrous for analysis), and sodium hydroxide (≥98%, pellets, anhydrous) were purchased from Sigma Aldrich (Germany). Bromine (99+%, extra pure) and hydroxy urea (98%) were from Thermo Scientific (Germany). The solvents used were purchased in HPLC grade (Table S1), except for ether, which was of technical grade.

Table S1: Additional information on the solvents used.

Solvent	Additional Information	Supplier
Dichloromethane	anhydrous, max. 0.001% water, stabilized with amylene	VWR Chemicals
Diphenyl ether	ReagentPlus®, ≥99%	Sigma-Aldrich
Ethyl acetate	for HPLC, ≥99.9%	Sigma-Aldrich
Methanol	for HPLC, ≥99.9%	Sigma-Aldrich
Tetrahydrofuran	anhydrous, ≥99.9%, inhibitor-free	Sigma-Aldrich

S1.2 Chromatography

Thin-layer chromatography was carried out with aluminum precast plates (0.25 mm silica 60, F254, Merck) for qualitative reaction control. Substances were visualized by staining with a potassium permanganate solution (4.00 g $KMnO_4$ and 2.00 g $NaHCO_3$ in 200 mL ultrapure water) followed by heat treatment. Silica gel Si 60 (230-240 mesh, particle size 40-63 μm) from Merck was used for column chromatography. The amount of silica gel and the column diameter depended on the separation problem. The composition of the eluent used is mentioned in the corresponding synthesis. The retention values R_f were determined by thin-layer chromatography.

S1.3 Nuclear Magnetic Resonance Spectroscopy

Nuclear magnetic resonance spectra (NMR) were recorded on the AVHD 400 and AVHD 500 from Bruker at 300 K. The chemical shifts δ are given in ppm and were reported in the ^{1}H

spectrum relative to the residual proton signals of the used deuterated solvent: $CDCl_3$, δ = 7.26 ppm, and DMSO-d_6, δ = 2.50 ppm. For the ^{13}C spectra, chemical shift information refers to the deuterium-coupled multiplets of the solvent used: $CDCl_3$, δ = 77.16 ppm, and DMSO-d_6, δ = 39.52 ppm. Multiplicities of the signals were given with the following abbreviations and their combinations: s = singlet, d = doublet, t = triplet, q = quartet, and m = multiplet. The reported coupling constants *J* correspond to the mean values of the experimentally found values in Hertz (Hz). ^{13}C-NMR spectra were recorded proton decoupled. The peaks were assigned based on findings from the literature and the resulting coupling of the introduced ^{15}N- and ^{13}C-labels.

S1.4 Characterization of Synthesis Products

3-Hyroxybutyronitrile (1a-c)

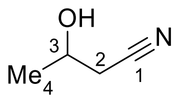

(**1a-c**)

R_f (ether): 0.67.

3-Hyroxybutyronitrile (**1a**) (3.39 g, 39.8 mmol, 77%):

^{1}H-NMR (500 MHz, $CDCl_3$): δ (ppm) = 1.36 (3 H, d, $^{3}J_{H\text{-}H}$ = 6.3 Hz, CH_3), 2.03 (1 H, s br, OH), 2.46-2.58 (2 H, m, CH_2), 4.14-4.20 (1 H, m, CH).

^{13}C-NMR (101 MHz, $CDCl_3$): δ (ppm) = 23.0 (CH_3), 27.6 (CH_2), 64.2 (CH), 117.7 (CN).

3-Hyroxybutyronitrile-3-^{13}C (**1b**) (3.56 g, 41.4 mmol, 80%):

^{1}H-NMR (500 MHz, $CDCl_3$): δ (ppm) = 1.36 (3 H, dd, $^{3}J_{H\text{-}H}$ = 6.2, $^{2}J_{H\text{-}C}$ = 4.5 Hz, CH_3), 1.90 (1 H, s br, OH), 2.47-2.58 (2 H, m, CH_2), 3.99-4.35 (1 H, m, ^{13}CH).

^{13}C-NMR (101 MHz, $CDCl_3$): δ (ppm) = 22.9 (d, *J* = 36.4 Hz, CH_3), 27.7 (d, *J* = 36.4 Hz, CH_2), 64.3 (m, ^{13}CH), 117.6 (CN).

3-Hyroxybutyronitrile-^{15}N (**1c**) (3.12 g, 36.2 mmol, 70%):

^{1}H-NMR (500 MHz, $CDCl_3$): δ (ppm) = 1.36 (3 H, d, $^{3}J_{H\text{-}H}$ = 6.1 Hz, CH_3), 2.11 (1 H, s br, OH), 2.47-2.57 (2 H, m, CH_2), 4.14-4.20 (1 H, m, CH).

^{13}C-NMR (101 MHz, $CDCl_3$): δ (ppm) = 22.9 (CH_3), 27.7 (CH_2), 64.3 (CH), 117.7 (d, *J* = 17.3 Hz, C^{15}N).

The ^{1}H- and ^{13}C-NMR spectra without using labeled starting material are in agreement with the literature.[1]

Crotononitrile (2a-c)

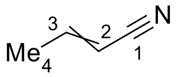

(**2a-c**)

Crotononitrile (**2a**) (54%, 546 mg, 8.15 mmol):

Z-isomer:

^{1}H-NMR (500 MHz, $CDCl_3$): δ (ppm) = 2.06 (3 H, dd, $^{3}J_{H\text{-}H}$ = 7.0 Hz, $^{4}J_{H\text{-}H}$ = 1.7 Hz, CH_3), 5.34-5.39 (1 H, m, C*H*-CH_3), 6.58 (1 H, dq, $^{3}J_{H\text{-}H}$ = 10.9 Hz, $^{3}J_{H\text{-}H}$ = 7.0 Hz, C*H*-CN).

^{13}C-NMR (101 MHz, $CDCl_3$): δ (ppm) = 17.6 (CH_3), 100.8 (*C*H-CN), 116.0 (CN), 150.1 (*C*H-CH_3).

E-isomer:

^{1}H-NMR (500 MHz, $CDCl_3$): δ (ppm) = 1.94 (3 H, dd, $^{3}J_{H\text{-}H}$ = 6.9 Hz, $^{4}J_{H\text{-}H}$ = 1.9 Hz, CH_3), 5.34-5.39 (1 H, m, C*H*-CH_3), 6.74 (1 H, dq, $^{3}J_{H\text{-}H}$ = 16.2 Hz, $^{3}J_{H\text{-}H}$ = 6.9 Hz, C*H*-CN).

^{13}C-NMR (101 MHz, $CDCl_3$): δ (ppm) = 19.2 (CH_3), 101.2 (*C*H-CN), 117.5 (CN), 151.4 (*C*H-CH_3).

Crotononitrile-3-^{13}C (**2b**) (38%, 390 mg, 5.74 mmol):

Z-isomer:

^{1}H-NMR (500 MHz, $CDCl_3$): δ (ppm) = 2.05 (3 H, td, $^{3}J_{H\text{-}H}$ = 7.1 Hz, $^{4}J_{H\text{-}H}$ = 1.8 Hz, CH_3), 5.32-5.37 (1 H, m, ^{13}C*H*-CH_3), 6.36-6.74 (1 H, m, C*H*-CN).

^{13}C-NMR (101 MHz, $CDCl_3$): δ (ppm) = 17.7 (d, J = 41.6 Hz, CH_3), 100.8 (d, J = 72.4 Hz, *C*H-CN), 116.0 (CN), 150.2 (*^{13}C*H-CH_3).

E-isomer:

^{1}H-NMR (500 MHz, $CDCl_3$): δ (ppm) = 1.92 (3 H, td, $^{3}J_{H\text{-}H}$ = 7.1 Hz, $^{4}J_{H\text{-}H}$ = 1.8 Hz, CH_3), 5.32-5.37 (1 H, m, ^{13}C*H*-CH_3), 6.53-6.92 (1 H, m, C*H*-CN).

^{13}C-NMR (101 MHz, $CDCl_3$): δ (ppm) = 19.3 (d, J = 41.6 Hz, CH_3), 101.2 (d, J = 72.8 Hz, *C*H-CN), 117.6 (CN), 151.4 (*^{13}C*H-CH_3).

Crotononitrile-^{15}N (**2c**) (35%, 359 mg, 5.29 mmol):

Z-isomer:

^{1}H-NMR (500 MHz, $CDCl_3$): δ (ppm) = 2.05 (3 H, dd, $^{3}J_{H\text{-}H}$ = 7.0 Hz, $^{4}J_{H\text{-}H}$ = 1.6 Hz, CH_3), 5.32-5.38 (1 H, m, C*H*-CH_3), 6.56 (1 H, dq, $^{3}J_{H\text{-}H}$ = 10.8 Hz, $^{3}J_{H\text{-}H}$ = 6.9 Hz, C*H*-C^{15}N).

^{13}C-NMR (101 MHz, $CDCl_3$): δ (ppm) = 17.7 (CH_3), 100.9 (*C*H-C^{15}N), 116.0 (d, J = 18.2 Hz, C^{15}N), 150.2 (*C*H-CH_3).

E-isomer:

^{1}H-NMR (500 MHz, $CDCl_3$): δ (ppm) = 1.92 (3 H, dd, $^{3}J_{H\text{-}H}$ = 6.9 Hz, $^{4}J_{H\text{-}H}$ = 1.8 Hz, CH_3), 5.32-5.38 (1 H, m, C*H*-CH_3), 6.73 (1 H, dq, $^{3}J_{H\text{-}H}$ = 16.3 Hz, $^{3}J_{H\text{-}H}$ = 6.9 Hz, C*H*-C^{15}N).

^{13}C-NMR (101 MHz, $CDCl_3$): δ (ppm) = 19.3 (CH_3), 101.3 (*C*H-C^{15}N), 117.5 (d, *J* = 18.2 Hz, C^{15}N), 151.3 (*C*H-CH_3).

The ^{1}H- and ^{13}C-NMR spectra without using labeled starting material are in agreement with the literature.[1]

3-Amino-5-methyl-isoxazole (3a-c)

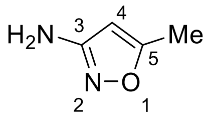

(**3a-c**)

3-Amino-5-methyl-isoxazole (**3a**) (77%, 2.22 g, 21.6 mmol):

^{1}H-NMR (500 MHz, DMSO-d$_6$): δ (ppm) = 2.20 (3 H, d, $^{4}J_{H\text{-}H}$ = 1.1 Hz, CH_3), 5.40 (2 H, s br, NH_2), 5.56 (1 H, d, $^{3}J_{H\text{-}H}$ = 0.9 Hz, CH).

^{13}C-NMR (101 MHz, DMSO-d$_6$): δ (ppm) = 11.9 (CH_3), 94.4 (CH), 164.0 (C-NH_2), 167.3 (C-O).

3-Amino-5-methyl-isoxazole-5-^{13}C (**3b**) (51%, 1.42 g, 14.3 mmol):

^{1}H-NMR (500 MHz, DMSO-d$_6$): δ (ppm) = 2.19 (3 H, dd, $^{2}J_{C\text{-}H}$ = 6.9 Hz, $^{4}J_{H\text{-}H}$ = 1.0 Hz, CH_3), 5.39 (2 H, s br, NH_2), 5.56 (1 H, dd, $^{2}J_{C\text{-}H}$ = 9.7 Hz, $^{4}J_{H\text{-}H}$ = 1.0 Hz, CH).

3-Amino-5-methyl-isoxazole-($^{15}NH_2$) (**3c**) (57%, 1.59 g, 16.0 mmol):

^{1}H-NMR (500 MHz, DMSO-d$_6$): δ (ppm) = 2.20 (3 H, d, $^{4}J_{H\text{-}H}$ = 0.9 Hz, CH_3), 5.39 (2 H, d, $^{1}J_{N\text{-}H}$ = 84.3 Hz, $^{15}NH_2$), 5.56 (1 H, s, CH).

^{13}C-NMR (101 MHz, DMSO-d$_6$): δ (ppm) = 11.9 (CH_3), 94.4 (CH), 164.0 (d, *J* = 16.5 Hz, C-$^{15}NH_2$), 167.3 (C-O).

The ^{1}H- and ^{13}C-NMR spectra without using labeled starting material are in agreement with the literature.[2]

4-Acetamidobenzene-1-sulfonyl chloride (4a-b)

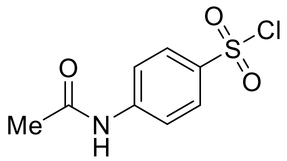

(**4a-b**)

4-Acetamidobenzene-1-sulfonyl chloride (**4a**) (87%, 3.44 g, 14.7 mmol):

^{1}H-NMR (500 MHz, DMSO-d$_6$): δ (ppm) = 2.03 (3 H, s, CH_3), 7.49 (4 H, m, CH_{ar}), 9.97 (1 H, s br, NH).

4-Acetamidobenzene-1-sulfonyl chloride-^{15}N (**4b**) (67%, 2.66 g, 11.3 mmol):

^{1}H-NMR (500 MHz, DMSO-d$_6$): δ (ppm) = 2.04 (3 H, s, CH_3), 7.49 (4 H, m, CH_{ar}), 9.97 (1 H, d, $^{2}J_{N\text{-}H}$ = 90.0 Hz, ^{15}NH).

Sulfamethoxazole (5a-d)

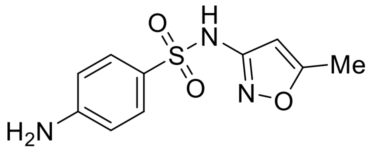

(**5a-d**)

Sulfamethoxazole (**5a**) (86%, 2.51 g, 10.3 mmol)

^{1}H-NMR (500 MHz, DMSO-d$_6$): δ (ppm) = 2.28 (3 H, d, $^{4}J_{H\text{-}H}$ = 0.9 Hz, CH_3), 6.07 (2 H, s br, NH_2), 6.08 (1 H, d, $^{4}J_{H\text{-}H}$ = 1.0 Hz, CH), 6.56-6.59 (2 H, m, CH_{ar}), 7.44-7.47 (2 H, m, CH_{ar}), 10.91 (1 H, s br, NH).

^{13}C-NMR (101 MHz, DMSO-d$_6$): δ (ppm) = 12.0 (CH_3), 95.3 (CH), 112.6 (C_{ar}), 124.2 (C_{ar}), 128.8 (C_{ar}), 153.3 (C_{ar}), 158.0 (-N-C=N-),169.8 (*C*-CH_3).

Sulfamethoxazole-($^{15}NH_2$) (**5b**) (48%, 1.46 g, 5.76 mmol)

^{1}H-NMR (400 MHz, DMSO-d$_6$): δ (ppm) = 2.28 (3 H, d, $^{4}J_{H\text{-}H}$ = 1.0 Hz, CH_3), 6.11 (2 H, d, $^{1}J_{N\text{-}H}$ = 87.3 Hz, NH_2), 6.08 (1 H, d, $^{4}J_{H\text{-}H}$ = 1.0 Hz, CH), 6.55-6.58 (2 H, m, CH_{ar}), 7.44-7.47 (2 H, m, CH_{ar}), 10.90 (1 H, s br, NH).

^{13}C-NMR (101 MHz, DMSO-d$_6$): δ (ppm) = 12.0 (CH_3), 95.3 (CH), 112.6 (C_{ar}), 124.1 (C_{ar}), 128.8 (C_{ar}), 153.3 (C_{ar}, d, J = 14.3 Hz), 158.0 (-N-C=N-), 169.8 (*C*-CH_3).

Sulfamethoxazole-(^{13}*C*-CH_3) (**5c**) (85%, 2.59 g, 10.2 mmol):

δ (ppm) = 2.28 (3 H, dd, $^{4}J_{H\text{-}H}$ = 1.0 Hz, $^{3}J_{H\text{-}C}$ = 7.0 Hz, CH_3), 6.06 (1 H, s br, NH_2), 6.08 (1 H, dd, $^{4}J_{H\text{-}H}$ = 1.1 Hz, $^{3}J_{H\text{-}C}$ = 9.6 Hz, CH), 6.56-6.60 (2 H, m, CH_{ar}), 7.44-7.48 (2 H, m, CH_{ar}), 10.90 (1 H, s br, NH).

^{13}C-NMR (101 MHz, DMSO-d$_6$): δ (ppm) = 12.0 (d, J = 50.7 Hz, CH_3), 95.3 (d, J = 70.7 Hz, CH), 112.6 (C_{ar}), 124.2 (C_{ar}), 128.8 (C_{ar}), 153.3 (C_{ar}), 158.0 (-N-C=N-), 169.8 (^{13}*C*-CH_3).

Sulfamethoxazole-(^{15}NH) (**5d**) (55%, 1.93 g, 7.59 mmol):

^{1}H-NMR (400 MHz, DMSO-d$_6$): δ (ppm) = 2.28 (3 H, m, CH_3), 6.05 (2 H, s br, NH_2), 6.08 (1 H, d, $^{4}J_{H\text{-}H}$ = 1.0 Hz, CH), 6.56-6.59 (2 H, m, CH_{ar}), 7.44-7.48 (2 H, m, CH_{ar}), 10.89 (1 H, s br, ^{15}NH).

^{13}C-NMR (101 MHz, DMSO-d$_6$): δ (ppm) = 12.0 (CH_3), 95.3 (CH), 112.6 (C_{ar}), 124.2 (C_{ar}, d, J = 3.5 Hz), 128.8 (C_{ar}), 153.3 (C_{ar}), 158.0 (-N-C=N-, d, J = 15.6 Hz), 169.8 (*C*-CH_3).

The ^{1}H- and ^{13}C-NMR spectra without using labeled starting material are in agreement with the literature.[3, 4] However, the ^{13}C-NMR spectrum of sulfamethoxazole-2-^{13}C revised the peak assignment of the two most downfield-shifted peaks.

S1.5 ^{1}H- and ^{13}C-NMR Spectra

3-Hyroxybutyronitrile-3-^{13}C (1b)

^{1}H-NMR (500 MHz, $CDCl_3$):

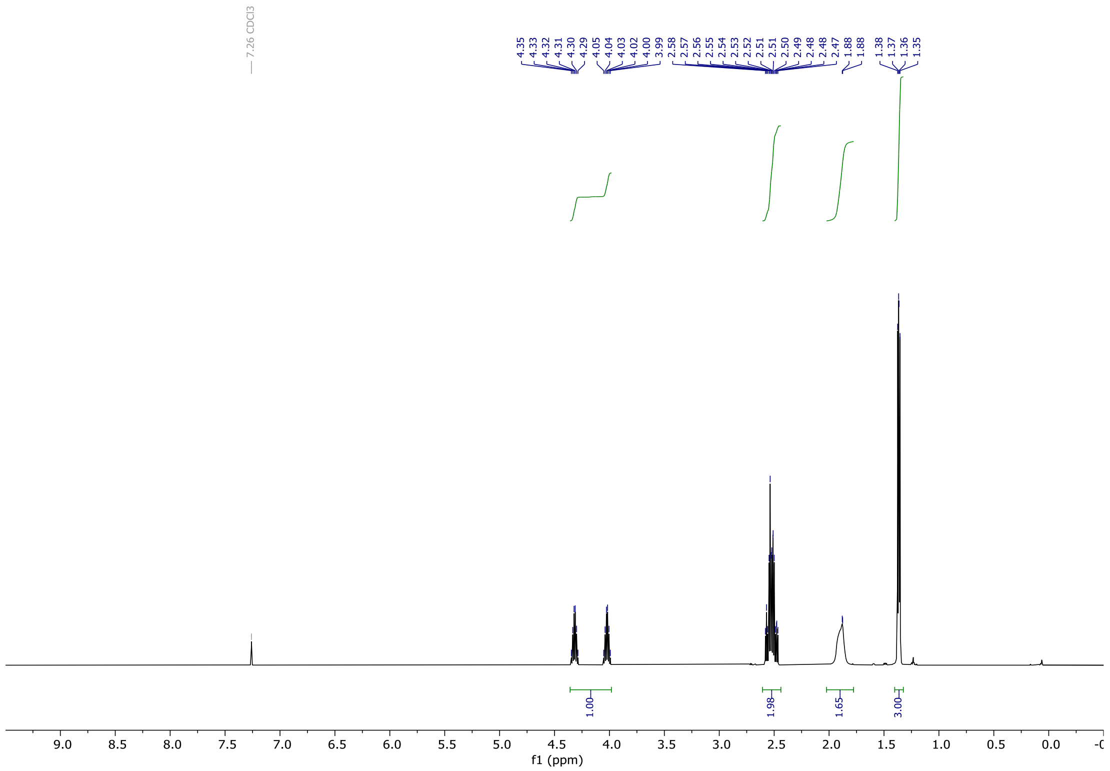

^{13}C-NMR (101 MHz, $CDCl_3$):

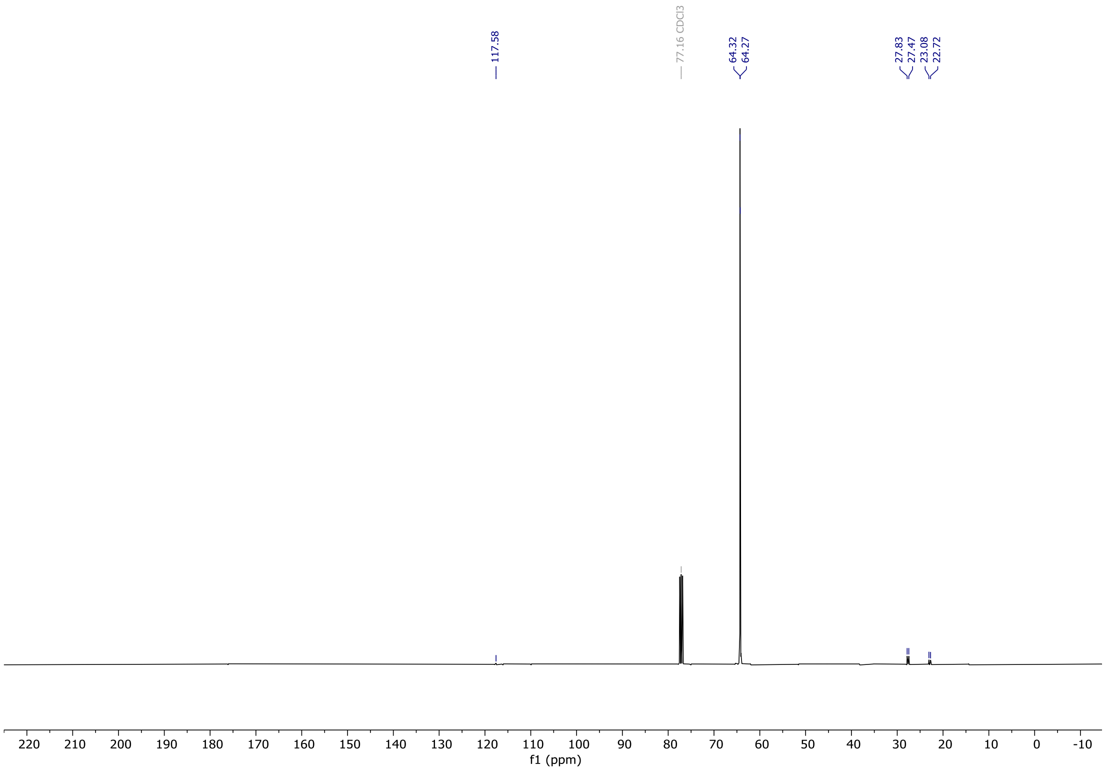

3-Hyroxybutyronitrile-^{15}N (1c)

^{1}H-NMR (500 MHz, $CDCl_3$):

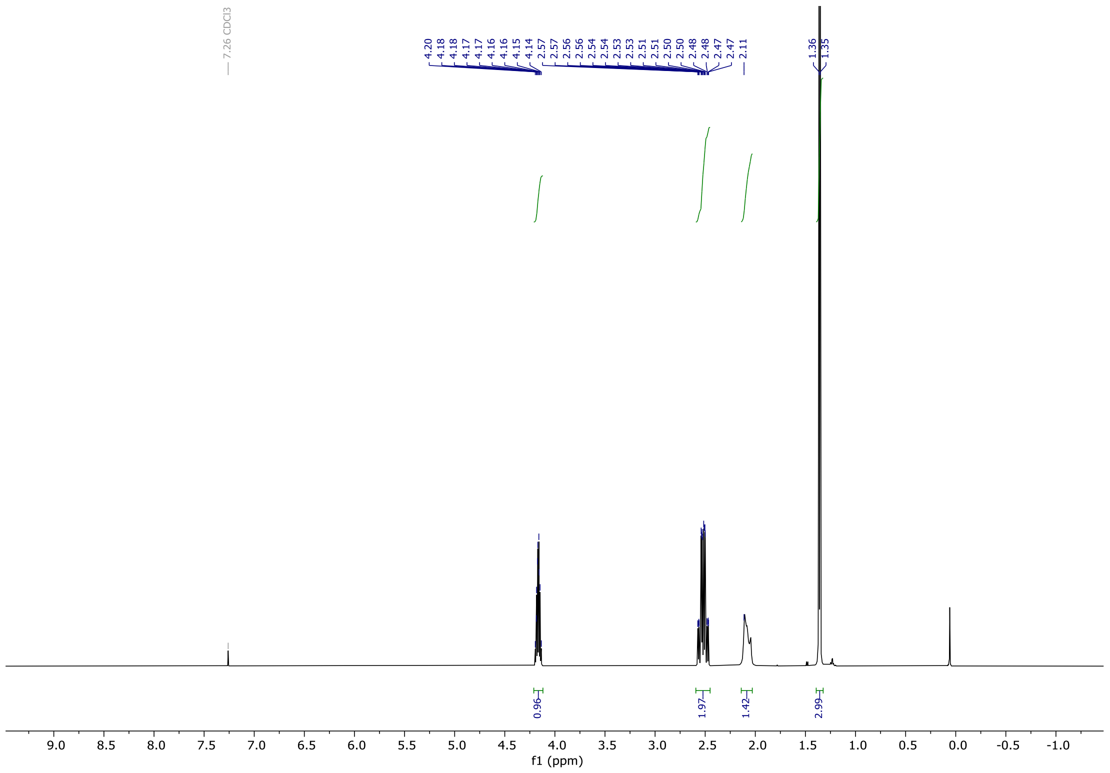

^{13}C-NMR (101 MHz, $CDCl_3$):

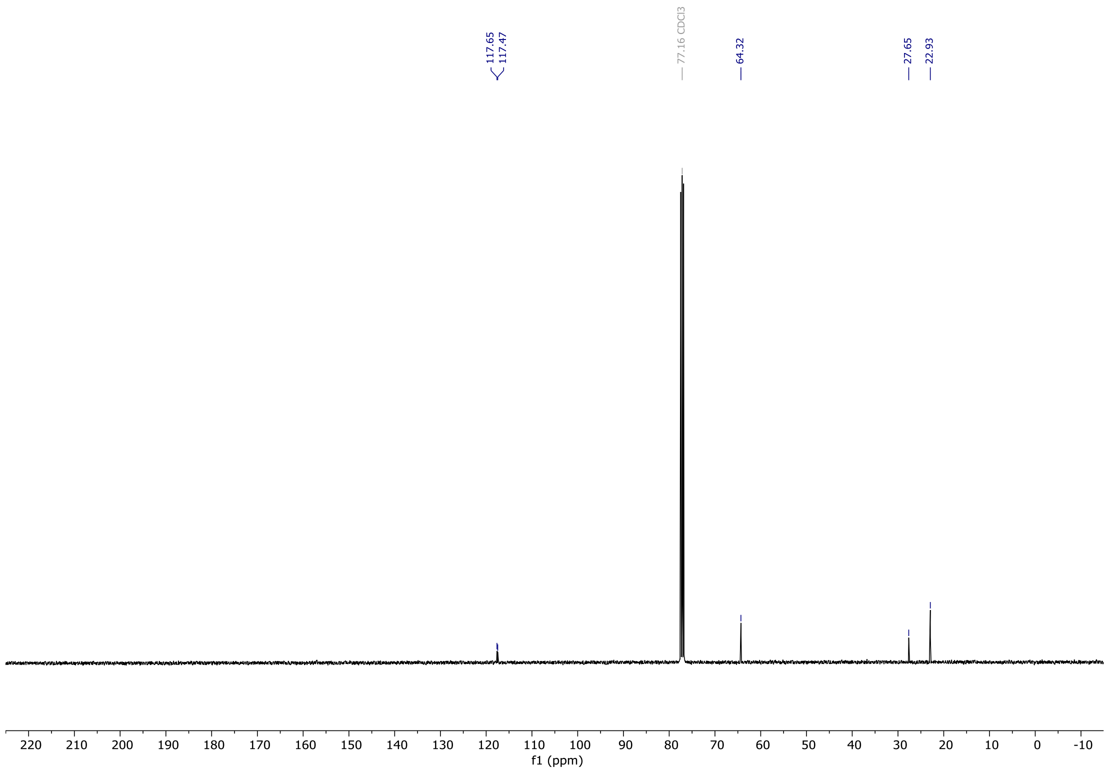

Crotononitrile-3-^{13}C (2b)

^{1}H-NMR (500 MHz, $CDCl_3$):

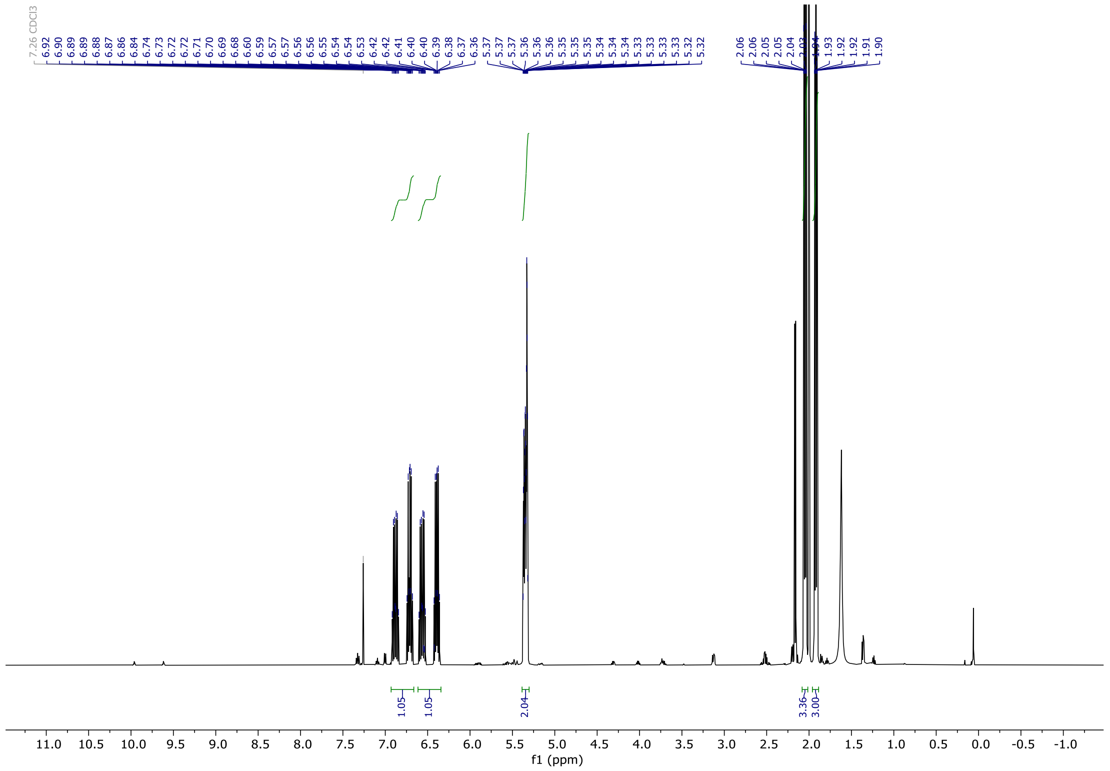

^{13}C-NMR (101 MHz, $CDCl_3$):

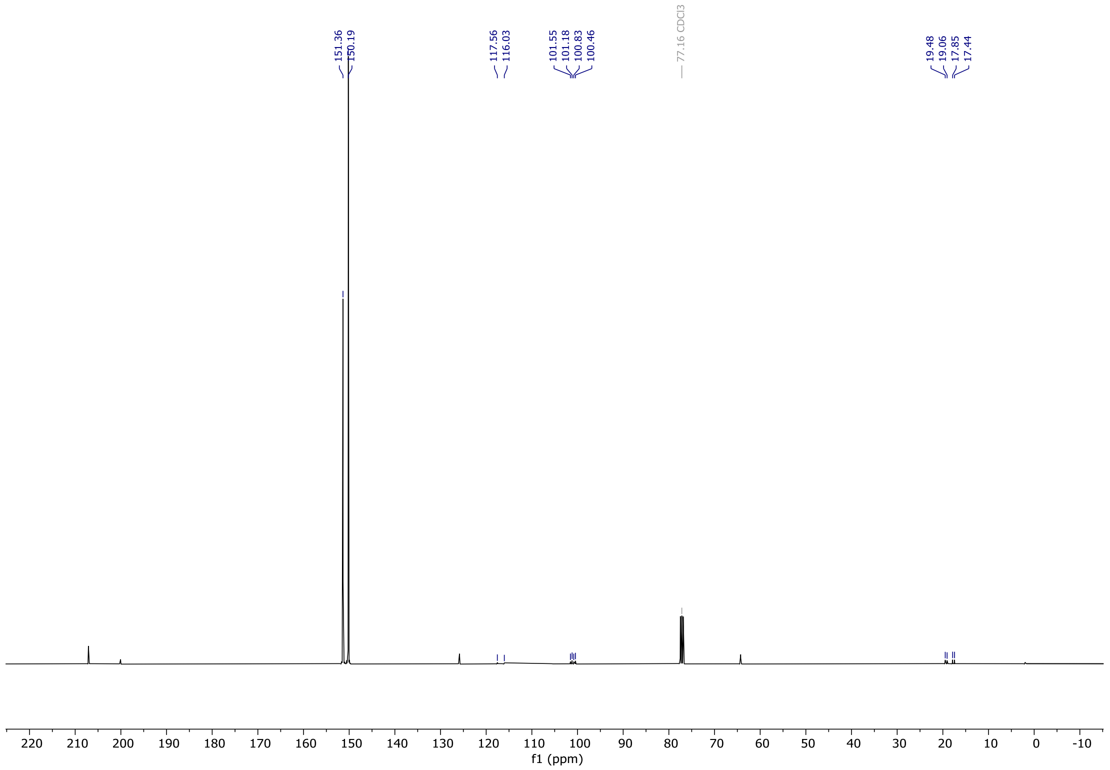

Crotononitrile-^{15}N (2c)

^{1}H-NMR (500 MHz, $CDCl_3$):

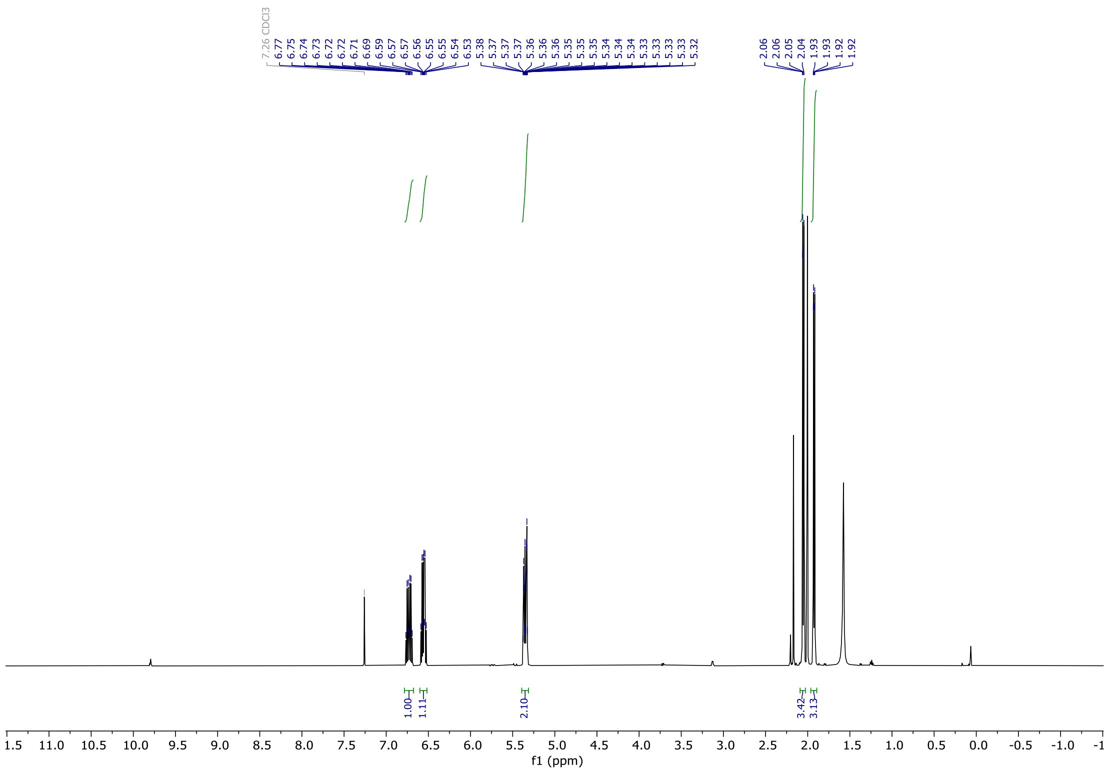

^{13}C-NMR (101 MHz, $CDCl_3$):

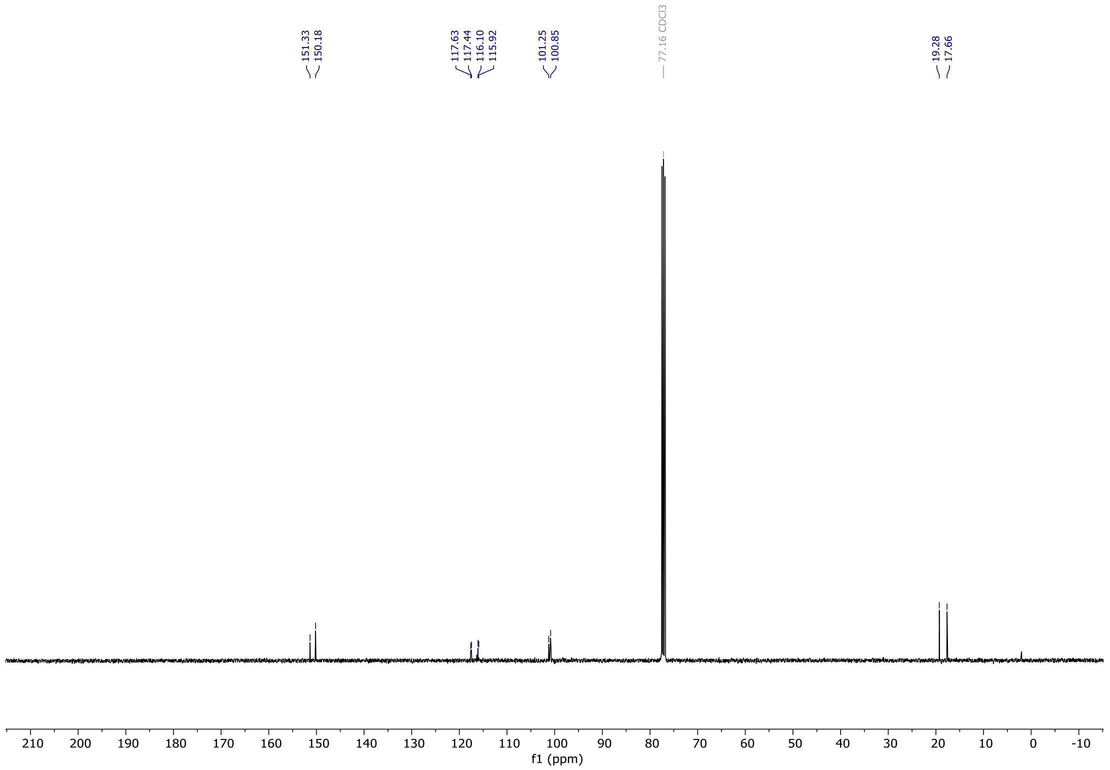

3-Amino-5-methyl-isoxazole-5-^{13}C (3b)

^{1}H-NMR (500 MHz, DMSO-d_6):

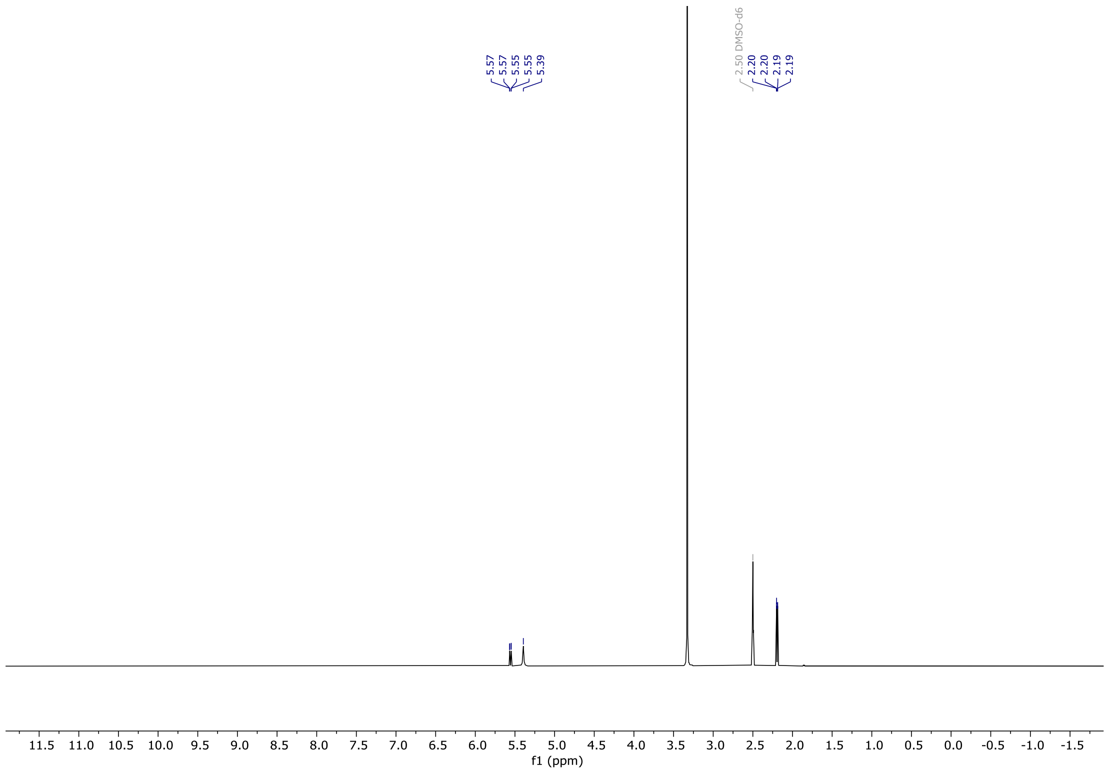

3-Amino-5-methyl-isoxazole-(^{15}NH$_2$) (3c)

^{1}H-NMR (500 MHz, DMSO-d_6):

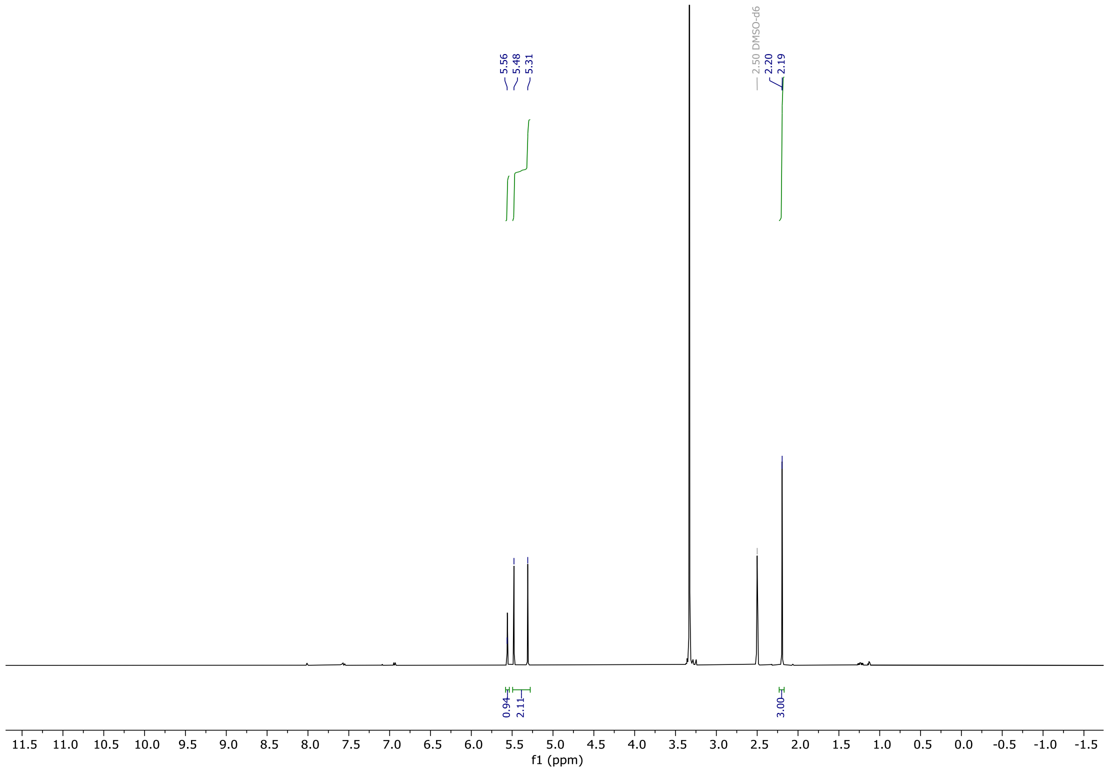

^{13}C-NMR (101 MHz, DMSO-d$_6$):

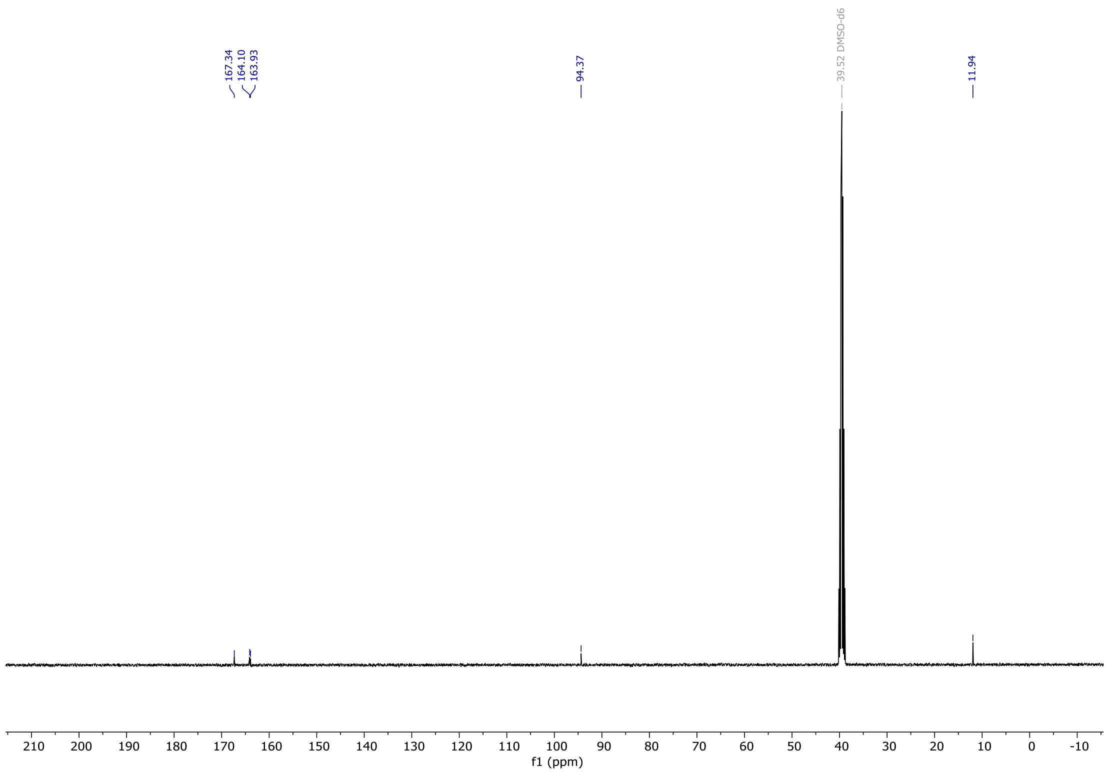

4-Acetamidobenzene-1-sulfonyl chloride-^{15}N (4b)

^{1}H-NMR (500 MHz, DMSO-d$_6$):

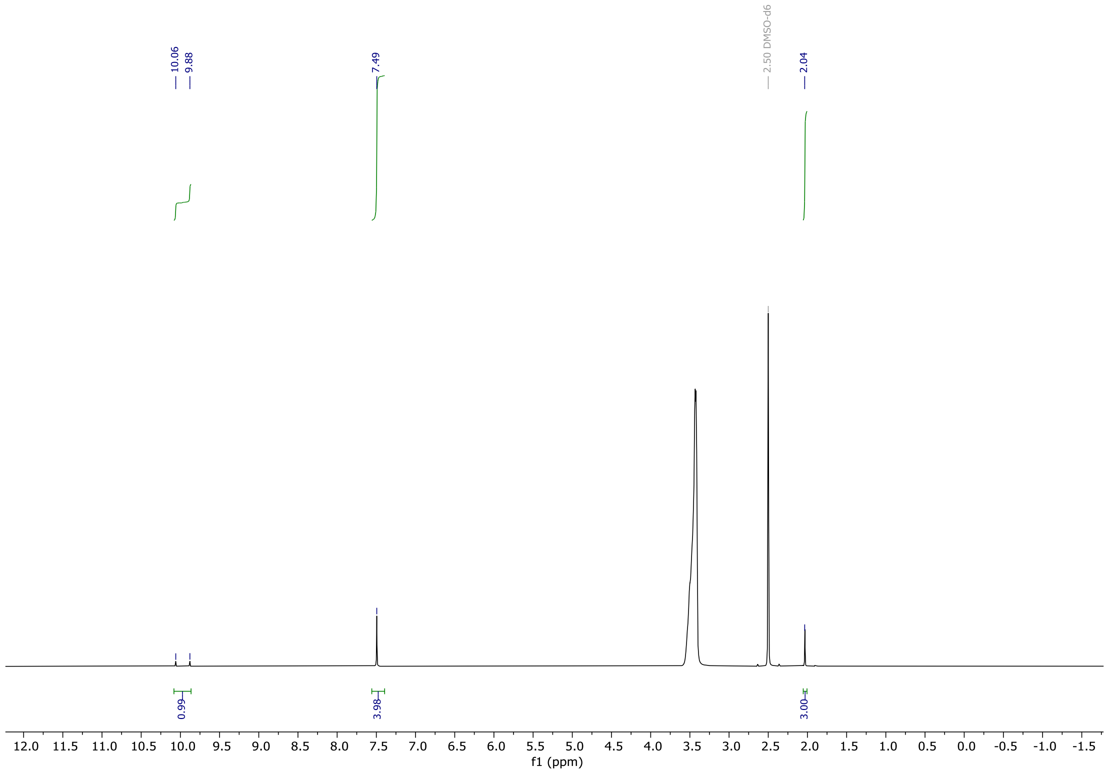

Sulfamethoxazole-($^{15}NH_2$) (5b)

^{1}H-NMR (400 MHz, DMSO-d$_6$):

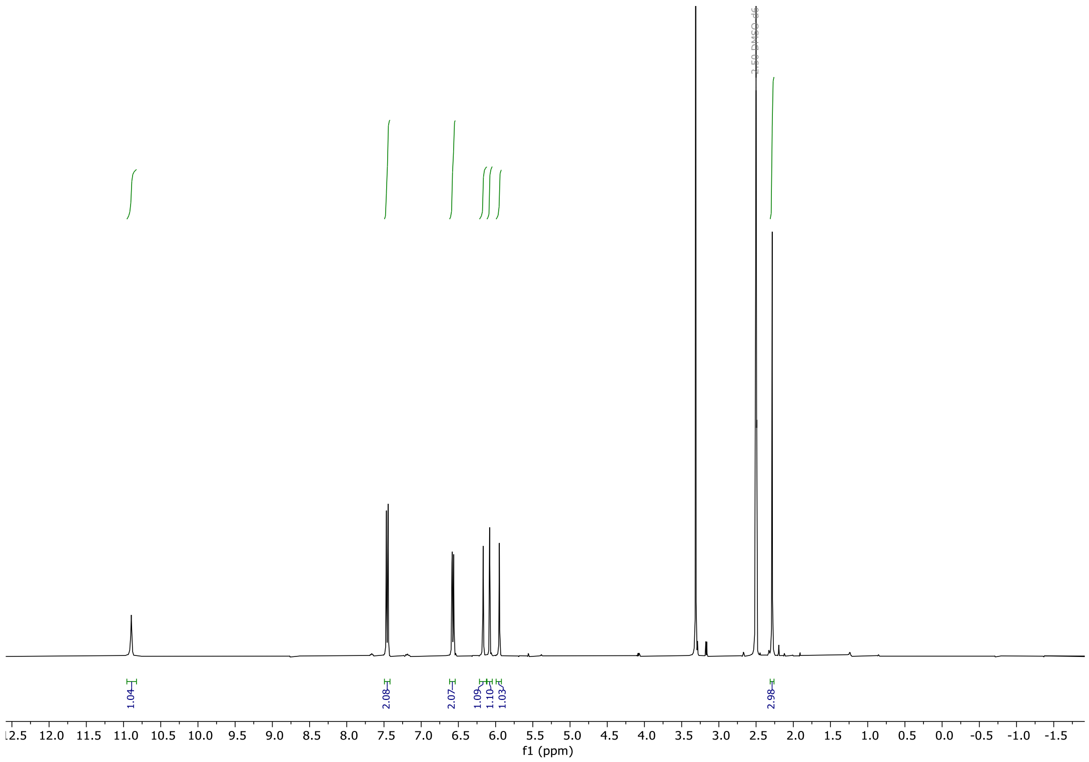

^{13}C-NMR (101 MHz, DMSO-d$_6$):

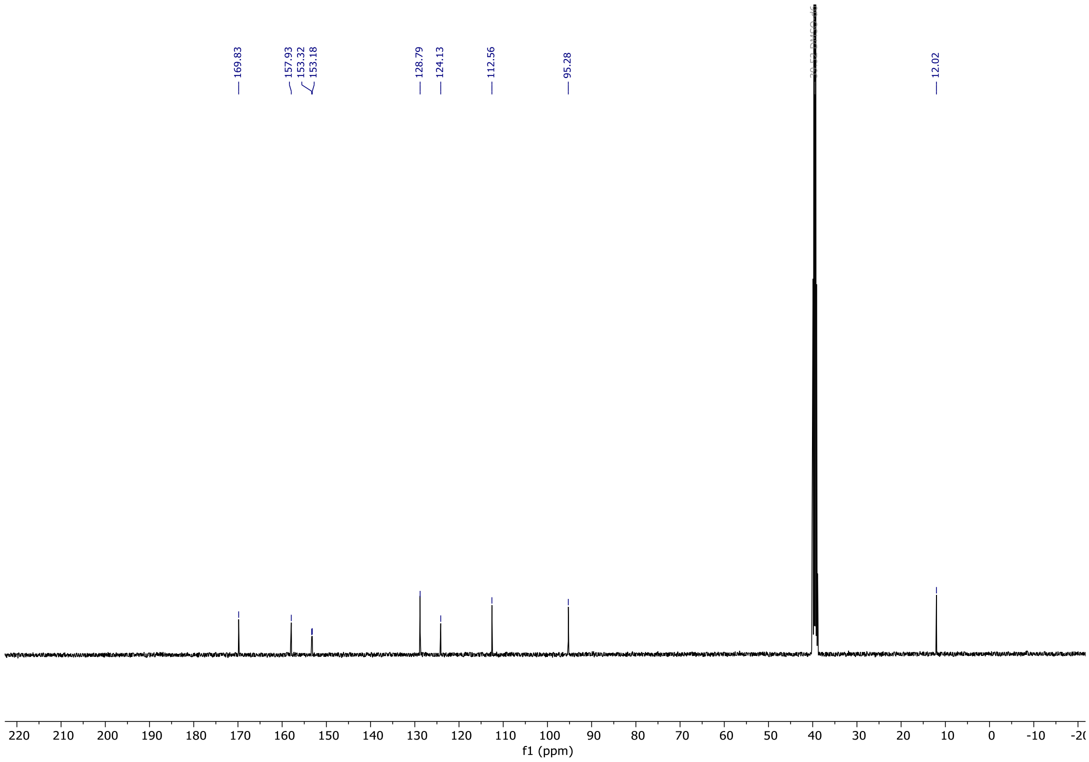

Sulfamethoxazole-(^{13}*C*-CH_3) (5c)

^{1}H-NMR (400 MHz, DMSO-d_6):

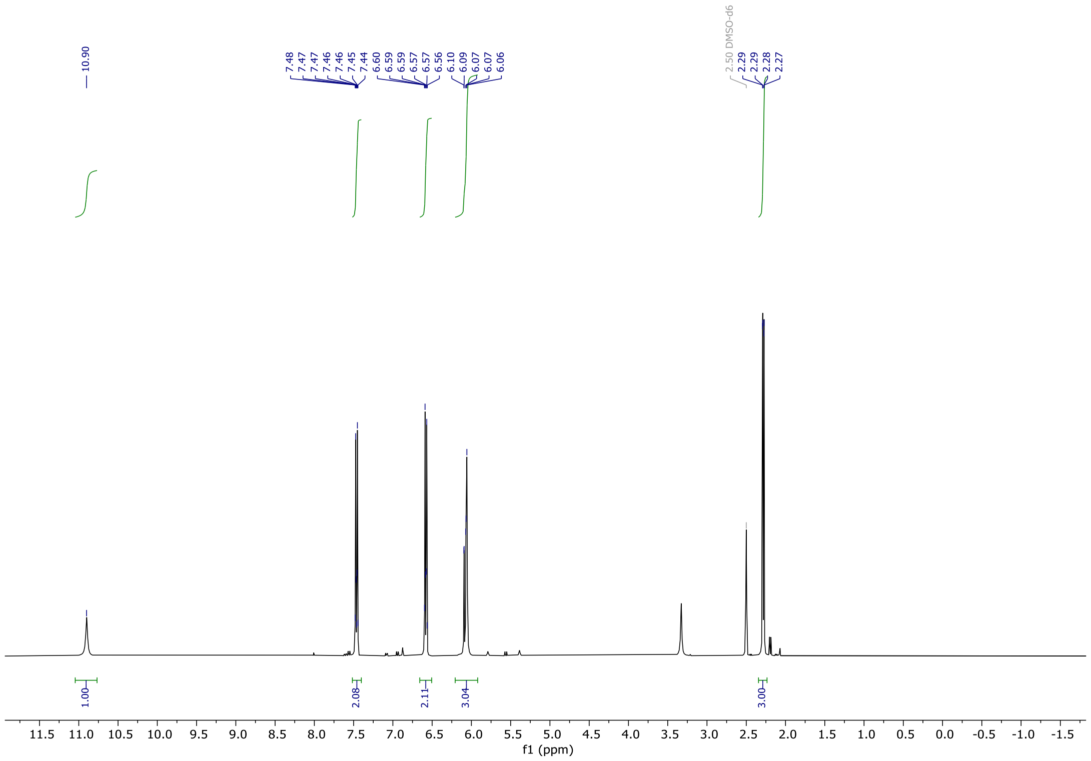

^{13}C-NMR (101 MHz, DMSO-d_6):

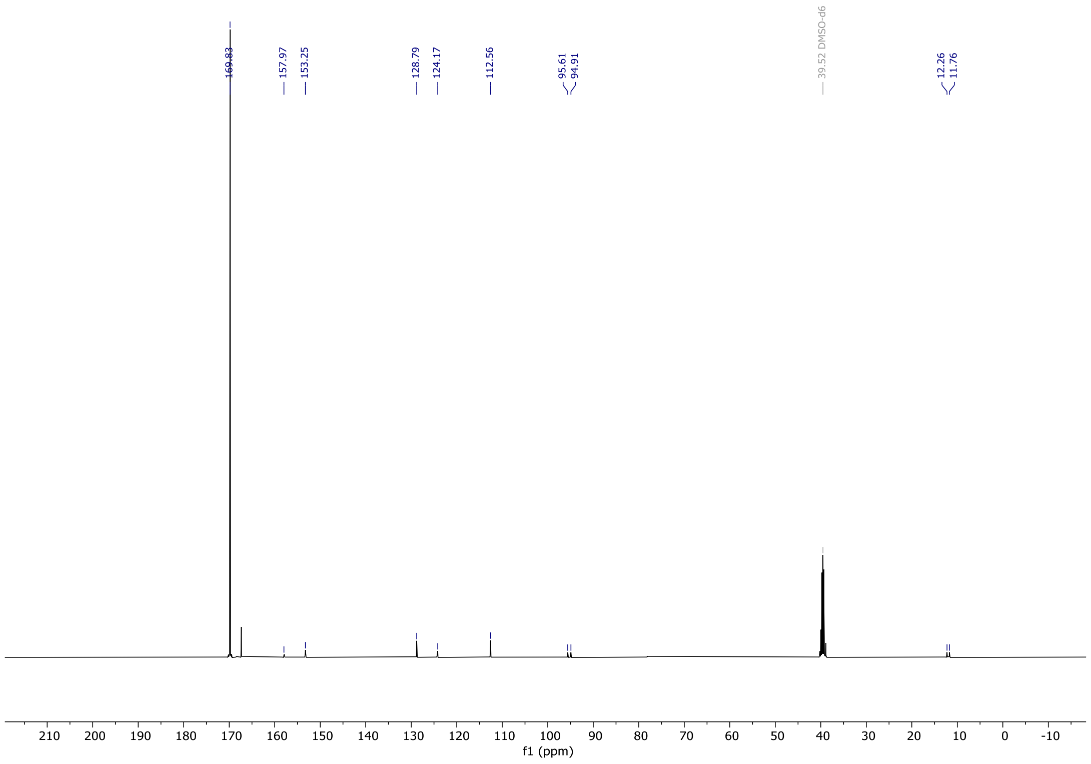

Sulfamethoxazole-(^{15}NH) (5d)

^{1}H-NMR (400 MHz, DMSO-d_6):

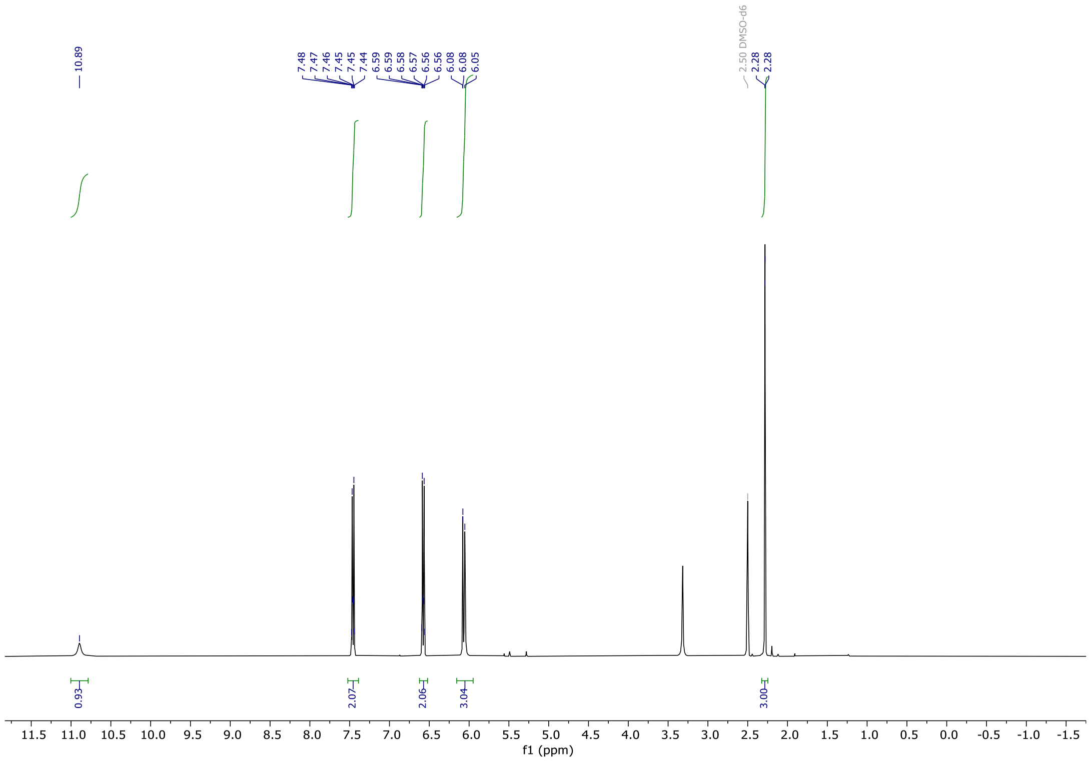

^{13}C-NMR (101 MHz, DMSO-d_6):

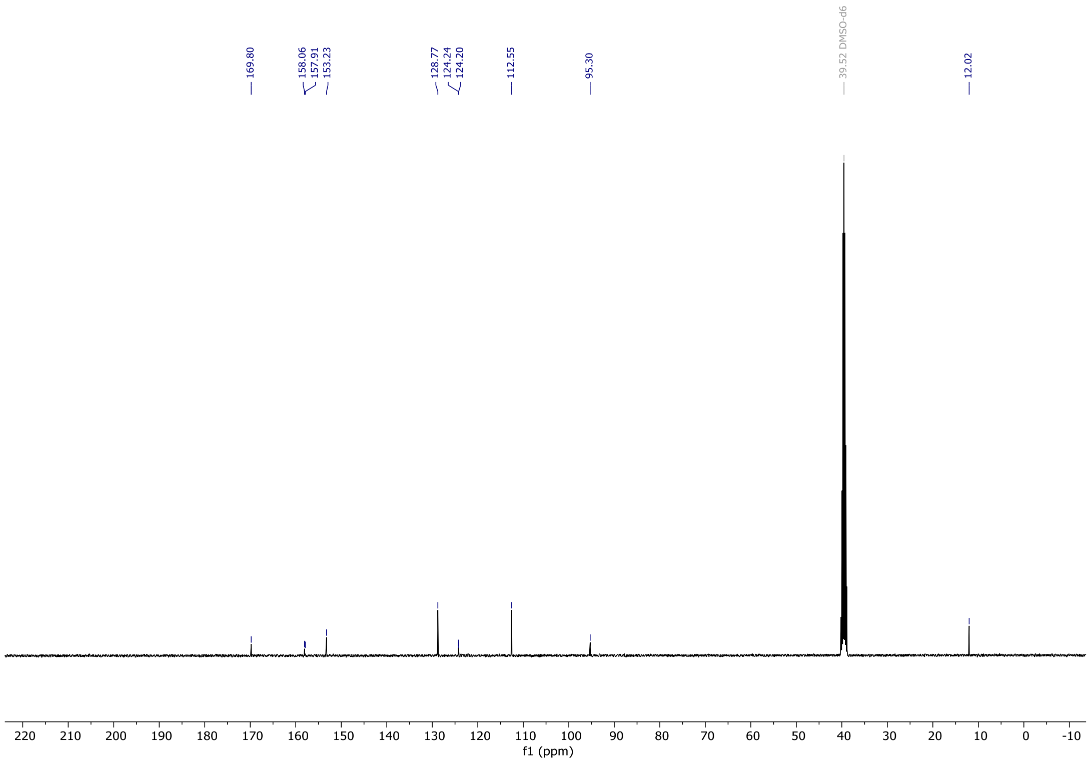

S2 Reversed-Phase Liquid Chromatography

The synthesis products **5b-d** were purified by HPLC on a Nexera XR HPLC system equipped with two solvent pumps (LC-20AD), an autosampler (SIL-20AC XR), a column oven (CTO-20A prominence), a diode array detector (SPD-M20A prominence), and a fraction collector (FRC-10A) (all, Shimadzu, Japan). A Gemini 5u C18 110A (particle size = 5 µm, L × D = 150 × 10.0 mm, Phenomenex, USA) was used for chromatographic separation while setting the column oven temperature to 40 °C and using a flow rate of 5 mL min^{-1}. As the mobile phase, a binary gradient consisting of water (A) and methanol (B) was used. The gradient started with 10% B for 1 min, then increased to 90% B within 9 min, where it was held at 90% B for 1 min. To this end, 45 µL of a ^{13}C-labeled SMX solution in methanol/water (~ 25 mg L^{-1}) was injected, and the fraction containing the ^{13}C-labeled SMX was collected. This procedure was repeated until ruffly the desired mass was collected.

S3 Elemental Analysis Isotope Ratio Mass Spectrometry

The determination of the carbon and nitrogen isotope compositions of our prepared SMX in-house standards was performed on an EA-IRMS system consisting of a EuroEA (Euro Vector, Italy), which was hyphenated to a Finnigan MAT 253 IRMS via a Finnigan ConFlo III interface (both, Thermo Fisher Scientific, Germany). The isotope ratios of samples were calibrated against the reference materials USGS40 (L-glutamic acid) and USGS41 (L-glutamic acid). The results are summarized in Table S2.

Table S2: Summary of $\delta^{13}C$ and $\delta^{15}N$ values from the prepared standards determined by EA-IRMS and $\Delta\delta^{13}C$ and $\Delta\delta^{15}N$ values for the target fragment, the error of the EA-IRMS measurements refers to 95% CIs and was propagated for the errors of $\Delta\delta^{13}C$ and $\Delta\delta^{15}N$.

Standard	$\delta^{13}C$ / ‰	$\delta^{15}N$ / ‰	$\Delta\delta^{13}C_{calc}$ / ‰	$\Delta\delta^{15}N_{calc}$ / ‰
SMX_0	-27.6 ± 0.2	0.0 ± 0.5	-	-
A1	-27.2 ± 0.2	15.0 ± 0.5	-	45.1 ± 0.7
A2	-27.7 ± 0.2	30.6 ± 0.5	-	92.0 ± 0.7
A3	-27.7 ± 0.2	60.4 ± 0.5	-	181.3 ± 0.7
B1	-21.7 ± 0.2	0.4 ± 0.5	14.8 ± 0.7	-
B2	-15.9 ± 0.2	0.4 ± 0.5	29.4 ± 0.7	-
B3	-3.8 ± 0.2	0.4 ± 0.5	59.5 ± 0.7	-
C1	-27.7 ± 0.2	23.9 ± 0.5	-	36 ± 1
C2	-27.6 ± 0.2	42.9 ± 0.5	-	64 ± 1
C3	-27.7 ± 0.2	86.4 ± 0.5	-	130 ± 1
D1	-20.2 ± 0.2	21.5 ± 0.5	18.5 ± 0.7	32 ± 1
D2	-15.8 ± 0.2	42.6 ± 0.5	29.5 ± 0.7	64 ± 1
D3	-3.8 ± 0.2	85.2 ± 0.5	59.5 ± 0.7	128 ± 1

S4 Reductive Transformation of Sulfamethoxazole

S4.1 Chemicals and Methods

Sulfamethoxazole (analytical standard, BCCH3594), acetonitrile (“HPLC-grade”), ethyl acetate (“HPLC-grade”), methanol (as Orbitrap eluent: “hypergrade for LC-MS”; as HPLC eluent: “HPLC-grade”), hydrochloric acid (puriss. p.a., ACS reagent, ≥37%), iron powder (nanopowder, 40-60 nm particle size, 99% trace metals basis), phosphoric acid (85%, for analysis), sodium chloride (ACS reagent, ≥99.0%), and sodium hydroxide (≥98%, pellets, anhydrous) were purchased from Sigma Aldrich (Germany); monosodium phosphate (≥99.0%) was from Fluka (Germany). Bayferrox 910 (goethite) was from Lanxess (Germany). The used water (18.2 MΩ cm at 25 °C) was from a Milli-Q® Reference water purification system (Merck Millipore, USA).

Working Under Anoxic Conditions. Anoxic experiments were performed inside a glovebox (nitrogen atmosphere, oxygen <0.5 ppm), and the used glassware was placed in the glovebox three days prior to use. For the anoxic experiments, the water was degassed by heating it to 60 °C and subsequently sparged with argon for three hours while vigorously stirring.

Preparation of an Fe(II) Solution. A Fe(II) stock solution was prepared by adding hydrochloric acid (37 wt%, 5.17 mL) and iron powder (1.82 g) to freshly degassed water (50 mL). The reaction mixture was subsequently stirred for 2 h at 80 °C. The light blue, grey suspension was filtered through a syringe filter (25 mm-PTFE-syringe filter, hydrophilic, 0.22 µm, BGB) inside the glovebox, resulting in a light blue solution.

S4.2 Transformation of Sulfamethoxazole using Fe(II) and Goethite

The degradation experiment of SMX with Fe(II) and goethite was conducted in 1 L Schott bottles under anoxic conditions, as described in the literature.[5] The final concentrations of the reactants were 8 g L^{-1} goethite, 0.5 mM Fe(II), 0.2 M NaCl, and 50 µM SMX at pH 7. The pH was adjusted throughout the experiment by manually adding either NaOH (1 M) or HCl (1 M) (7 ± 0.2). The water at pH 7, goethite, and the Fe(II) solution were mixed, and the pH of the suspension was adjusted to 7 with 1 M hydrochloric acid and 1 M sodium hydroxide. After stirring the suspension overnight to equilibrate the Fe(II)/goethite system, the reaction was initiated by adding an SMX solution (0.25 mM, 220 mL). The reaction suspension was continuously stirred in the dark. After 2 h, 67 mL of the suspension was quenched by the addition of sodium hydroxide to app. pH 12 inside the glovebox, followed by subsequent filtration (25 mm-PTFE-syringe filter, hydrophilic, 0.22 µm, BGB) outside the glovebox.[6]

Afterward, 1 mL was used for concentration analysis, and the remaining sample was concentrated using solid-phase extraction (SPE).

S4.3 Solid-Phase Extraction of Sulfamethoxazole

The sample's pH was adjusted to 3.7, and solid-phase extraction (SPE) was performed using Oasis HLB cartridges (6 mL, 200 mg; Waters, Milford, MA, USA), following the procedure described by Liu et al.[7]. The cartridges were preconditioned by drop-wise percolation of acetonitrile (5 mL), followed by ethyl acetate (5 mL), and then water (5 mL) through the cartridges. Next, the samples were dropwise percolated through the cartridges and dried under vacuum overnight. The SMX was eluted from the cartridges with ethyl acetate (5 mL) followed by acetonitrile (5 mL). The eluate was dried under a gentle stream of nitrogen (0.5 L min^{-1}, 25 °C, TurboVap®, Biotage, Sweden) to complete dryness and reconstituted in 500 µL of methanol. These methanol extracts were diluted with water to achieve a target concentration of 100 µM for fragment-specific stable isotope analysis using ESI-Orbitrap-MS.

S4.4 GC-IRMS Measurements

Carbon Isotope Analysis. The $\delta^{13}C$ isotope analysis was performed on a Trace 1310 gas chromatograph connected via a GC IsoLink II and a ConFlo IV interface to a Finnigan MAT 253 isotope ratio mass spectrometer (all, Thermo Fisher Scientific, Germany). Therefore, 1.5 µL of the samples were injected by a TriPlusRSH (Thermo Fisher Scientific, Germany) into a split/splitless injector equipped with a splitless liner (4 mm ID × 78.5 mm length, 800 µL, ultra inert, double taper, without glass wool, Agilent Technologies, USA). The injector was operated in a split mode employing a split ratio of 1:3 at 280 °C. For the separation of the target analyte, an Agilent DB-1 column (30 m × 0.32 mm × 1.00 µm, Agilent Technologies, USA) with pre- and post-column (each 1 m deactivated fused silica guard column, 0.32 mm ID, Agilent, USA) with a constant helium flow rate of 2.0 mL min^{-1} was used with a GC-oven starting temperature of 170 °C, increasing to 320 °C with a ramp of 20 °C min^{-1} and a hold time of 5 min. After elution, the eluates underwent oxidation at a temperature of 1000 °C in a commercial combustion reactor (NiO tube CuO-NiO reactor, 2 mm, Thermo Fisher, Germany). The reactor was oxidized for 15 s after each injection to ensure consistent oxidation conditions. The limits of precise isotope analysis were determined using varying SMX concentrations (2 - 20 nmol C on column) of an in-house standard. Retention times and isotope values were checked regularly. Samples were measured in triplicate and bracketed with an in-house standard of SMX and USGS62 to monitor system stability. The reproducibility of the IRMS measurements remained below the total error of 0.5‰ for $\delta^{13}C$ values.

Nitrogen Isotope Analysis. Before GC-IRMS measurements for nitrogen stable isotope analysis, SMX was derivatized according to Dou and Canavan et al.[8]. Briefly, 900 µL of methanol and 100 µL of a SMX extract were added to a 20 mL crimp cap vial. Next, 160 µL 1.8 M-2.4 M trimethylsilyldiazomethane (TMSD, Thermo Fisher Scientific, Germany) in hexanes was added, and the vial was tightly closed. After placing the vial in a water bath at 50 °C for 1 h, the solvent was evaporated under a gentle stream of nitrogen (0.5 mL min^{-1}, 25 °C, TurboVap®, Biotage, Sweden) and the residue was reconstituted in methanol (267 µL). The stable nitrogen isotope analysis was performed on a GC-IRMS consisting of a Trace GC Ultra connected via Finnigan GC Combustion III interface to a Finnigan MAT 253 mass spectrometer (all, Thermo Fisher Scientific, Germany). The system was equipped with a customized oxidation reactor consisting of an alumina tube (320 mm length, 0.5 mm ID, 1.5 mm OD, Elemtex, UK) enclosing two nickel wires (0.1 mm diameter, 99.994% purity, Alfa Aesar, Germany) and one platinum wire (0.1 mm diameter, 99.997% purity, Thermo Fisher Scientific, Germany), and a customized reduction reactor filled with three copper wires (0.1 mm diameter, 99.9999% purity, Alfa Aeser, Germany). The samples were injected by a GC PAL autosampler (3 µL, CTC Analytics AG, Switzerland) into a split/splitless injector equipped with a splitless liner (5 mm ID x 106 mm length, Agilent, USA) maintained at a temperature of 250 °C. Splitless mode was used for the first minute with a surge pressure of 250 kPa, and afterwards switched to split mode with a split flow rate of 20 mL min^{-1}. An Agilent J&W DB-5MS UI column (30 m length x 0.25 mm ID x 1.0 µm film thickness, Agilent Technologies, USA) with pre- and post-column (each 1 m deactivated fused silica guard column, 0.25 mm ID, Agilent, USA) with a constant helium flow rate of 1.4 mL min^{-1} was used for the separation of the compounds. The GC oven was initially held at 120 °C for 1 min, increased to 250 °C with a ramp of 22 °C min^{-1}, and further increased to 325 °C with a ramp of 40 °C min^{-1}, where it was held for 9 min. After elution, the eluates underwent oxidation at a temperature of 1030 °C, followed by a reduction at 650 °C to remove unwanted nitrogen oxides. To ensure consistent oxidation, the reactor was oxidized for 20 min and subsequently held in backflush for 20 min to remove any undesired oxidation products. A liquid nitrogen trap preceded the entry of the gases into the IRMS to capture the CO_2 generated during combustion. This ensured that CO_2 did not enter the ion source, preventing any potential interference with the measurement of N_2 isotopologues. The trap was emptied daily by removing the liquid N_2 during reoxidation of the reactor. Retention times and isotope values were monitored regularly, samples were measured in duplicate and bracketed with an in-house standard of freshly derivatized SMX to monitor system stability. The reproducibility of the IRMS measurements remained below the total error of 1.0‰ for $\delta^{15}N$ values. The limit of isotope analysis and the

methods' trueness were measured using varying concentrations of an in-house standard of SMX (4 - 16 nmol N on column) for derivatization and subsequent isotope analysis. The software Isodat (Thermo Fisher Scientific, Germany) automatically performed peak detection and integration using the individual background algorithm for baseline correction of both carbon and nitrogen isotope analysis.

S5 Concentration Analysis

Concentration analysis of SMX-containing samples was performed on the same HPLC system, as described in S2. SMX was separated on a RP18 column (XTerra RP18 Column, length: 150 mm, diameter: 3.0 mm, particle size: 3.5 µm, Waters, USA) at 40 °C with a flow rate of 0.5 mL min^{-1}. A binary gradient with mobile phase A (water with 0.1 % formic acid) and mobile phase B (methanol) was used. Eluent B was kept at 10% for 1 min and increased to 90% over the next 8 min, where it was held for 1 min until the portion of B was reduced to 10% over 2 min. The concentration of SMX was determined by creating and measuring calibration standards at a wavelength of 268 nm. The limit of quantification (LOQ) was determined in accordance with DIN 32645. Data processing was performed by LabSolutions, version 5.117 (Shimadzu, Japan).

S6 References

(1) Boers, R. B.; Randulfe, Y. P.; Haas, H. N. v. d.; Rossum-Baan, M. v.; Lugtenburg, J. Synthesis and spectroscopic characterization of 1-^{13}C-and 4-^{13}C-plastoquinone-9. *Eur. J. Org. Chem.* **2002**, *2002* (13), 2094–2108.
(2) Iwai, I.; Nakamura, N. Studies on acetylenic compounds. XLIV. Synthesis of 3-aminoisoxazoles and 3-hydroxyisoxazoles (3-isoxazolones). *Chem. Pharm. Bull.* **1966**, *14* (11), 1277–1286.
(3) Sunduru, N.; Salin, O.; Gylfe, Å.; Elofsson, M. Design, synthesis and evaluation of novel polypharmacological antichlamydial agents. *Eur. J. Med. Chem.* **2015**, *101*, 595–603.
(4) Zhou, W.; Moore, D. E. Photochemical decomposition of sulfamethoxazole. *Int. J. Pharm.* **1994**, *110* (1), 55–63.
(5) Mohatt, J. L.; Hu, L.; Finneran, K. T.; Strathmann, T. J. Microbially Mediated Abiotic Transformation of the Antimicrobial Agent Sulfamethoxazole under Iron-Reducing Soil Conditions. *Environ. Sci. Technol.* **2011**, *45* (11), 4793–4801.
(6) Elsner, M.; Haderlein, S. B.; Kellerhals, T.; Luzi, S.; Zwank, L.; Angst, W.; Schwarzenbach, R. P. Mechanisms and products of surface-mediated reductive dehalogenation of carbon tetrachloride by Fe (II) on goethite. *Environ. Sci. Technol.* **2004**, *38* (7), 2058–2066.
(7) Liu, X.; Akay, C.; Kopke, J.; Kummel, S.; Richnow, H. H.; Imfeld, G. Direct Phototransformation of Sulfamethoxazole Characterized by Four-Dimensional Element Compound Specific Isotope Analysis. *Environ. Sci. Technol.* **2024**, *58* (23), 10322–10333.
(8) Dou, Q.; Canavan, A.; Fu, Y.; Xiang, L.; Wang, Y.; Wang, X.; Jiang, X.; Dirr, C.; Wang, F.; Elsner, M. Nitrogen stable isotope analysis of sulfonamides by derivatization-gas chromatography-isotope ratio mass spectrometry. *Anal. Bioanal. Chem.* **2024**, *416* (19), 4237–4247.
